# Exploring the landscape of symbiotic diversity and distribution in unicellular ciliated protists

**DOI:** 10.1186/s40168-024-01809-w

**Published:** 2024-05-24

**Authors:** Bing Zhang, Liwen Xiao, Liping Lyu, Fangqing Zhao, Miao Miao

**Affiliations:** 1 University of Chinese Academy of Sciences, Beijing, 100049 China; 2 Institute of Zoology, Beijing Institutes of Life Science, Chinese Academy of Sciences, Beijing, 100101 China; 3 Key Laboratory of Systems Biology, Hangzhou Institute for Advanced Study, University of Chinese Academy of Sciences, Hangzhou, China; 4 Key Laboratory of Evolution & Marine Biodiversity (Ministry of Education), and Institute of Evolution and Marine Biodiversity, Ocean University of China, Qingdao, 266003 China

## Abstract

**Background:**

The eukaryotic-bacterial symbiotic system plays an important role in various physiological, developmental, and evolutionary processes. However, our current understanding is largely limited to multicellular eukaryotes without adequate consideration of diverse unicellular protists, including ciliates.

**Results:**

To investigate the bacterial profiles associated with unicellular organisms, we collected 246 ciliate samples spanning the entire Ciliophora phylum and conducted single-cell based metagenome sequencing. This effort has yielded the most extensive collection of bacteria linked to unicellular protists to date. From this dataset, we identified 883 bacterial species capable of cohabiting with ciliates, unveiling the genomes of 116 novel bacterial cohabitants along with 7 novel archaeal cohabitants. Highlighting the intimate relationship between ciliates and their cohabitants, our study unveiled that over 90% of ciliates coexist with bacteria, with individual hosts fostering symbiotic relationships with multiple bacteria concurrently, resulting in the observation of seven distinct symbiotic patterns among bacteria. Our exploration of symbiotic mechanisms revealed the impact of host digestion on the intracellular diversity of cohabitants. Additionally, we identified the presence of eukaryotic-like proteins in bacteria as a potential contributing factor to their resistance against host digestion, thereby expanding their potential host range.

**Conclusions:**

As the first large-scale analysis of prokaryotic associations with ciliate protists, this study provides a valuable resource for future research on eukaryotic-bacterial symbioses.

Video Abstract

**Supplementary Information:**

The online version contains supplementary material available at 10.1186/s40168-024-01809-w.

## Background

The symbiotic relationship between bacteria and eukaryotes is believed to have played a significant role in the evolution and speciation [1]. Evolutionarily ancient endosymbionts are believed to be the origin of eukaryotic organelles such as mitochondria and chloroplasts, and the ongoing symbionts have been reported to offer a range of benefits to the host including metabolism [2], nutritional replenishment [3], defense [4], and mobility [5]. Even facultative symbionts of arthropods, such as Wolbachia, has been found to manipulate the reproductive properties of its hosts [6]. Research on the eukaryotic-bacterial symbiotic relationships has revealed a spectrum of symbiotic interactions that range from facultative to obligate (which may or may not be mutualistic) [7] and from transient to permanent and stable. Some bacteria have been found to establish intracellular niches within both human cells and unicellular protists, which are evolutionarily distant hosts. Notable examples include *Legionella pneumophila* [8, 9], *Pseudomonas aeruginosa* [10, 11], *Francisella novicida* [12], *Coxiella burnetii* [13], and *Mycobacterium avium* [14]. Ciliates constitute a highly diverse group of protists, with over 8000 documented free-living species [15], and their evolutionary history can be traced back to approximately 1.1 billion years [16]. Given their remarkable species diversity, widespread distribution, and diverse nutritional preferences [17–20], ciliates have emerged as an ideal model for studying fundamental life processes in eukaryotes [21–25].

Traditionally, intracellular bacteria in ciliates have been termed “symbionts” [26]. However, microscopic and genomic studies have revealed a wide array of bacterial communities inhabiting the cell surface and compartments of intracellular ciliates [27]. Recent studies have also demonstrated that a *Rickettsiales* bacterium can replicate independently on *Paramecium* surface [28], challenging the previous notion of their obligate intracellular nature. Another bacterial endosymbiont, “*Candidatus* Azoamicus ciliaticola,” provides energy through denitrification to its anaerobic ciliate host, representing an intermediate stage of evolution towards becoming an organelle [29]. However, due to the challenges associated with culturing symbionts independently, the regulatory mechanisms underlying the establishment and maintenance of endosymbiotic relationships remain poorly understood. Previous studies on a limited number of successfully cultured symbionts have provided insights into certain genes and pathways contributing to the symbiotic system [30–35]. However, these studies only represent a limited fraction of the vast ciliate-bacterial interactions present in natural environments. To achieve a comprehensive understanding of the prevalence and significance of these symbiotic relationships, further research encompassing a wide range of ciliate species and environmental conditions is necessary.

In this study, we leveraged metagenome-based techniques to characterize the cohabitating bacterial profiles of 246 specimens from 91 species of 12 classes at an unprecedented detail. Based on the 3.1 Tbp genomic sequence data, we identified 6,042,995 bacterial-derived contigs. We have provided evidence through metagenomic studies that bacteria are commonly found within ciliates, shedding light on distinct bacterial patterns observed among different ciliate species. Extensive analyses were conducted to investigate the composition patterns, functional characteristics, and influencing factors related to bacteria interacting with ciliates, which revealed the significance of eukaryotic-like proteins in bacterial infection of the host. These findings provided a significant contribution to the understanding of the evolution and function of the eukaryotic cohabitants.

## Results

### Large-scale discovery of ciliate-associated bacteria

**Fig. 1:**
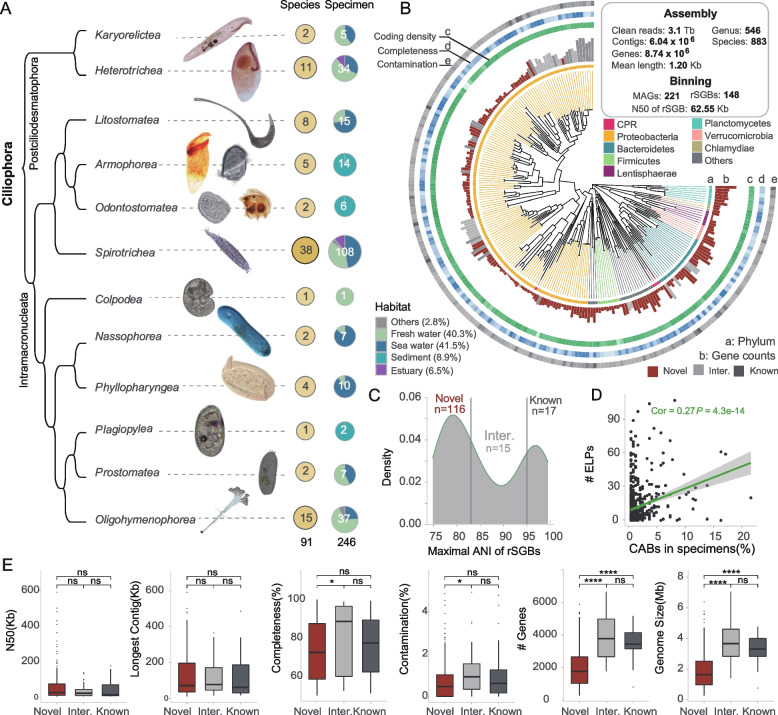
Identification of ciliate-associated bacteria from samples covering almost the entire phylum Ciliophora. **A** Specimens covers almost the entire Ciliophora phylum. **B** Approximately maximum-likelihood phylogenetic tree of the 148 rSGBs in ciliates. The circular regions labeled from a to e in the plot represent the taxonomy of phylum, gene number, density of coding genes, completeness, and contamination, respectively. **C** Distribution of the maximum ANI distance of rSGBs compared to the reference database. **D** Spearman correlation analysis between the number of ELPs in CABs and their frequency in specimens. **E** A comparison of the genome size, N50, longest contig, completeness, contamination, gene content and genome size among different types of rSGBs. All *P* values in this figure were calculated based on two-tailed Wilcoxon test

To comprehensively investigate the diversity of ciliate-associated bacteria (CABs), we have collected the most complete cohort of ciliates known to date (Suppl. Table S1), which covers 12 classes under the phylum Ciliophora, with only 2 marine anaerobic classes (*Muranotrichea* and *Parablepharismea*) not being included. The sampling environments included freshwater, marine water, brackish water, and sediment, as well as eight anaerobic ciliates. To ensure comprehensive retrieval of CABs, we applied two widely-used genome amplification methods [36] in biological replicates of each species. In total, we generated 246 sequencing datasets for 91 species from 81 genera (Fig. 1A, Suppl. Table S1). After trimming of sequencing adapters and low-quality sequences, we generated 10.4 billion paired-end reads with a total of 3.1 Tbp data.

Given the limited availability of studied genomes for most ciliates, we adopted an assembly-based strategy to remove potential host sequences. Then, the decontaminated reads were reassembled into contigs using Megahit [37] and classified using MMseqs2 [38], which generated 6,042,995 bacterial-derived contigs with an average length of 1197 bp (Suppl. Figure S1A). On average, the proportion of bacterial sequences in each specimen was 4.44% (Suppl. Figure S1B). To assess the efficacy of bacterial discrimination, we plotted the distribution of contigs’ GC content against their sequencing depth and found a distinct separation between the host- and bacterial-derived contigs (*P* < 2.2e−16, Wilcoxon test), but no difference was observed between host-derived and randomly selected contigs (*P* > 0.05, Wilcoxon test) (Suppl. Figure S1C-D). We further employed another tool Kaiju to verify the accuracy of contig classification, which utilizes translated sequences for comparison with a reference database of microbial proteins [39]. As shown in Suppl. Figure S3E, 78.2% of bacterial-derived contigs were supported by both methods.

To quantify the unknown bacteria within CABs, we examined the taxonomy of contigs that were longer than 1 kb. Given the extensive studies on human gut bacteria, we utilized a metagenomic dataset consisting of ten human fecal samples as a control. Notably, the proportion of unclassified contigs in ciliate samples was two to three times higher compared to the human fecal samples (Suppl. Figure S1F). It is worth noting that due to the challenges associated with assembling low biomass bacteria, the actual proportion of unknown taxa should even be underestimated. This observation suggests a significant gap in our understanding of ciliate-associated bacteria.

To characterize the composition of CABs, we classified bacterial-derived contigs and identified a total of 546 genera and 883 bacterial species with the cutoff of total contig length >100 kb in a specimen (Fig. 1B). After binning contigs, by filtering bins and clustering them with average nucleotide identity (ANI) greater than 95%, we obtained 148 species-level clusters. For each cluster, we selected a representative single genomic bin (rSGB) based on maximum completeness and minimum contamination. Next, we compared the consistency between 148 rSGBs with the NCBI Refseq database to determine their highest similarity to known species (called maximum ANI) (Fig. 1C, Suppl. Table S2). Novelty assessment based on the maximum ANI followed commonly accepted criteria: a rSGB with a maximum ANI >95% was regarded as known species, the value <83% as novel species, and the value in between as intermediate [40]. In total, 78.2% of the rSGBs were classified to be novel bacteria (Fig. 1C).

Overall, more than half of rSGBs belonged to the *Proteobacteriota* phylum (Suppl. Figure S3A-B), while only seven belonged to the *Rickettsiales* order and five belonged to the phylum of *Verrucomicrobia* (Suppl. Figure S3A-B). Among these rSGBs, we found an endosymbiont of the *Polynucleobacter* genus in *Euplotes woodruffi*, which had the closest phylogenetic relationship with *P. yangtzensis*, and the ANI was only 84% (Suppl. Figure S2A). We further identified three novel species belonging to the class *Syntrophorhabdia* in anaerobic ciliates (Suppl. Figure S2B-D). The *Syntrophorhabdia* class is known for their ability to degrade aromatic pollutants, achieved through syntrophic associations with H2-consuming partner organisms [41, 42]. However, only ten genomes of this class have been published. The cohabitants we uncovered had a maximum ANI of 74% with the type species of this class (Suppl. Figure S2B-D). Additionally, certain species of *Legionella* genus are known for their intracellular pathogenicity in humans [43], with ciliates being identified as one of the possible hosts for *Legionella* [44]. We identified three distinct endosymbiotic *Legionella* bacterium (Suppl. Figure S2E-F). Particularly intriguing is the rSGB136 (Suppl. Figure S2E-F), with a genome size of less than 1 Mb. This cohabitant exhibited similar characteristics in terms of completeness, contamination, and genome size, when compared to previously reported species of the Coxiella-like endosymbiont of *Ambyolomma* and *Rhipicephalus*. Phylogenetic analysis showed that rSGB136 belongs to the family *Coxiellaceae* (Suppl. Figure S2E-F), which contains a variety of intracellular parasitic bacteria capable of infecting arthropods, ruminants, amoebas, and occasionally humans [45].

### Typical symbiotic characteristics of CABs

We have identified a considerable number of CABs at both the contig and metagenome-assembled genome (MAG) levels, surpassing the extent of previous studies [46–48]. However, their exact origin and the precise nature of relationship with ciliates remain uncertain. The presence of CABs solely at the contig level indicates that these bacteria exhibit lower abundance in comparison to CABs identified at the MAG level. To explore the relationship between ciliates and those lower abundance CABs, we have noted the potential involvement of eukaryotic-like proteins (ELPs) in mediating bacterial endosymbiosis within eukaryotes. ELPs are a class of proteins found in prokaryotes that share similarities in sequence features or structural domains [49]. ELPs possess domains normally exclusive to eukaryotes and have been proposed to facilitate pathogenic relationships between bacteria and their hosts [9, 50, 51]. The overexpression of ELPs from an uncultured *Gammaproteobacteria* symbiont of sponge in *Escherichia coli* increased its survival in amoebic phagosomes [51, 52], thereby highlighting the role of ELPs in interfering with the maturation of host phagosomes. Based on those studies, we hypothesized that bacteria with a higher content of ELPs may exhibit enhanced survival against host phagosomal digestion and, consequently, higher infection rates.

To validate this hypothesis, we retrieved complete genomes of 883 CABs species from NCBI and performed functional annotation using the eggNOG-mapper for 3,498,163 genes [53]. We identified 8422 ELPs (Suppl. Figure S3C), which could be categorized into eukaryotic orthogroups. They exhibited a widespread distribution in 73.5% of CABs, with a median of six ELPs per CAB (Suppl. Figure S3D). Functional analysis based on KEGG BRITE database revealed that these ELPs are mainly involved in membrane trafficking, peptidases and inhibitors, exosomes, ion channels, and gpi−anchored proteins, all of which contribute to the disruption of host phagosome maturation (Suppl. Figure S3E). Notably, we observed a significant positive correlation between the number of ELPs and the infection rate of the host (Spearman cor. = 0.27, *P* = 4.3e−14) (Fig. 1D). However, the number of ELPs did not correlate with the abundance of CABs (Spearman cor. = 0.01, *P* = 0.78) (Suppl. Figure S3F). We further confirmed these findings by correcting for the total number of genes in the bacteria (Suppl. Figure S3G-H). These results confirm that ELPs may play a key role in helping bacteria evade host phagosomes but may not contribute to their amplification within host.

To investigate the relationship between ciliates and CABs at the MAG level, we observed that endosymbiotic bacteria often undergo extensive genome reduction, resulting in smaller genomes and gene content [54, 55]. Accordingly, through the analysis of 148 rSGBs, we found no statistically significant differences in the genome assembly quality, as assessed by N50 and the longest contig, across the novel, known, and intermediate rSGBs (*P* > 0.05, Wilcoxon test) (Fig. 1E). Similarly, there were no significant differences in genome completeness and contamination rates between the novel and known rSGBs (*P* > 0.05, Wilcoxon test) (Fig. 1E). However, the genome size and gene content of the novel rSGBs were notably smaller compared to other rSGBs (*P* < 0.001, Wilcoxon test) (Fig. 1E). These results suggest that the bacteria we identified at the rSGB level may maintain a long-term endosymbiotic relationship with their hosts. Meanwhile, during specimen collection, *E. coli* or *Klebsiella pneumoniae* was utilized as food to maintain ciliate activity. These bacteria can serve as spike-in controls for assessing the presence of incompletely digested bacteria. Notably, the genomic bins of these food bacteria were only detected in 9.76% of the ciliate samples, indicating that most of the rSGBs are likely symbiotic bacteria capable of thriving and proliferating in cohabitation with ciliates. On the other hand, *E. coli* has been reported to establish an intracellular symbiosis with *Tetrahymena pyriformis* [56, 57], and transient symbiosis might be one of the reasons for its presence in the samples.

### Phenotypic and compositional characteristics of CABs

**Fig. 2:**
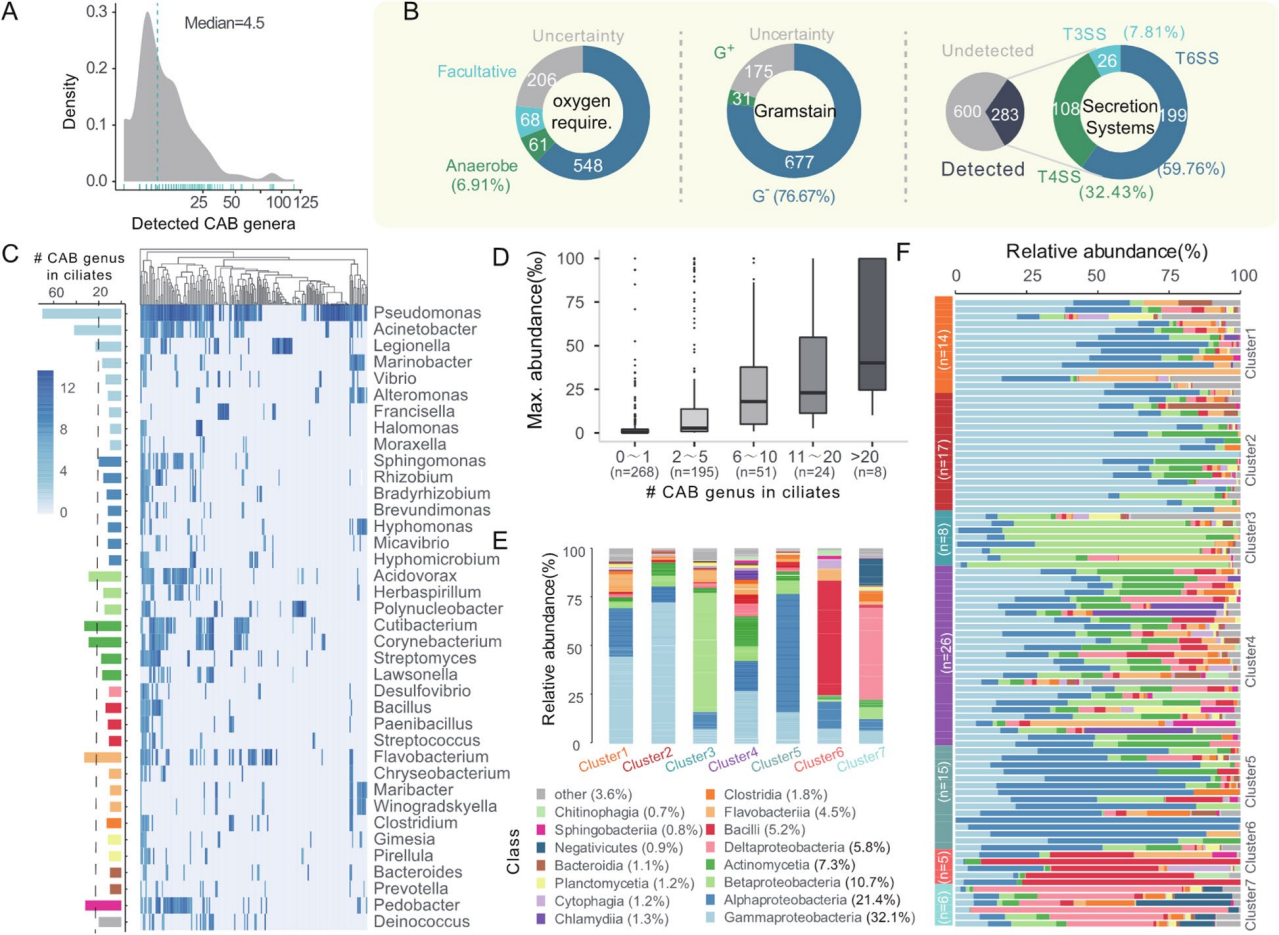
The composition and characteristics of the ciliate-associated bacteria (CAB). **A** Distribution of CABs genera among 246 specimens. Each vertical bar denotes one specimen. **B** Phenotype characteristics of 883 ciliate-associated species. The panels from left to right illustrate the following features: aerobic type, Gram stain type, and secretion system, respectively. **C** Distribution of symbiotic bacteria at the genus level in 91 ciliate species. Dashed line and red font indicate the most common symbiotic bacteria identified in more than 20 ciliate species. The heat map represents the average abundance of symbionts in each ciliate. **D** Distribution of maximum abundance of symbiotic bacteria at the genus level in 91 ciliates. **E–F** Seven clustering groups of ciliate symbiotic bacteria based on their abundance profiles. The bar diagram shows the distribution of CABs at the class level

After determining the symbiotic role of CABs, we proceeded to analyze their distribution and composition in different ciliates. Overall, more than 90% of the ciliates were found to coexist with CABs (Suppl. Figure S3I), with an average of approximately four different bacteria per ciliate cell (Fig. 2A and Suppl. Figure S3J). It should be noted that there may be some risk of slight overestimation as 24% of samples contained 2 to 3 host cells.

Phenotypic analysis using PhenDB [58] and MacSyFinder [59] revealed that the majority of the 883 CABs species were aerobic and Gram-negative bacteria (Fig. 2B). Previous studies have highlighted the role of type III, IV, and VI secretion systems (T3SS, T4SS, and T6SS, respectively) in symbiotic interactions, as they can disrupt phagosome maturation or suppress host defenses [60–63]. Our results showed that T6SS (59.7%) was the most detected secretion system in CABs, followed by T4SS and T3SS (Fig. 2B). However, these secretion systems were only present in 32.05% of CABs. Considering that some secretion systems are encoded by plasmids [64] and the assembled genome are incomplete and fragmented, the actual number of secretion systems may be underestimated. When comparing the abundance of CABs in the host and the infection rate, we found that CABs harboring those secretion systems exhibited a higher infection rate than those lacking (*P* = 0.045, Wilcoxon test) (Suppl. Figure S4A). However, there was no significant difference between the two groups in terms of abundance (*P* = 0.55, Wilcoxon test) (Suppl. Figure S4B). In addition, 39 CABs species have been reported as symbionts and 20 are phototrophic bacteria (Suppl. Table S3).

In terms of CABs composition, *Proteobacteriota* was the most predominant phylum among the 546 CABs genera, representing over half of the total composition (54.58%), followed by *Bacteroidota* (9.17%) and *Firmicuteota* (4.64%) (Suppl. Figure S4C-D), which is consistent with the observation in ciliate *Stentor coeruleus* [47]. Although *Proteobacteriota* and *Firmicutes* are both common bacteria, contrasting trends have been reported in ciliates and ciliate-inhabited environments [47]. Among the classes, *Alphaproteobacteria* (32.1%), *Gammaproteobacteria* (21.4%), and *Betaproteobacteria* (10.7%) were the most abundant (Suppl. Figure S4C).

Our findings also suggest a broader host range for several CABs. For example, a bacteria of “*Candidatus* Dependentiae” [65], “*Candidatus* Finniella inopinata” [66], and *Microcystis aeruginosa* [67], previously reported in amoebas, have also been located in nine, four, and six species of ciliates, respectively. Similarly, *Caedimonas*, an endosymbiont of *Paramecium* [4], was detected in samples from three distinct ciliates (Suppl. Figure S4E-F, Table S3). Moreover, certain genera like *Pseudomonas*, *Acinetobacter*, *Legionella*, *Acidovorax*, *Cutibacterium*, *Corynebacterium*, *Flavobacterium*, and *Pedobacter* were found in over 20 species of ciliates (Fig. 2C). *Polynucleobacter*, a genus associated with *Euplotes*, was identified in 15 ciliate species across 28 samples (Suppl. Figure S4E-F). When evaluated at the genus level, we found that 50.9% of CABs had more than two ciliate hosts (Fig. 2D). Within the 883 identified species, a significant positive correlation was observed between the diversity of hosts and the maximum bacterial abundance that could be achieved within the host (Pearson cor. = 0.44; *P* < 2.22e−16).

### Influential factors of the ciliate-associated bacterial community

**Fig. 3:**
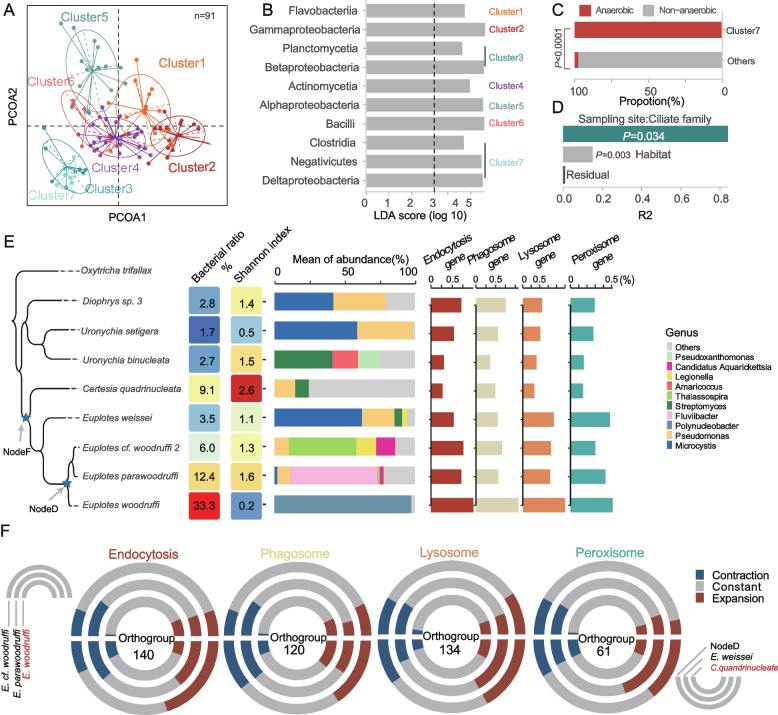
Influence of host, environment, and genomic amplification methods on microbial community. **A** Visualization of the PCoA and clustering analysis of class-level symbiotic communities among 91 ciliate species. **B** The signature bacterial class of CABs cluster. LDA score determined using linear discriminant analysis effect size (LEfSe) analysis. **C** The barplot illustrates the significant enrichment of Cluster 7 in anaerobic ciliates. **D** Distribution of R2 values for different factors in the analysis of the CABs community of 91 ciliates using the Permanova test. Each bar represents the R2 values for a specific factor, indicating the extent to which the factor explains the variation in the composition of CABs. **E** The influence of the host on CABs. From left to right: phylogenetic tree of *Euplotes*, total bacterial content, Shannon index, symbiont relative abundance at the genus level, and content of functional genes. **F** Gene family expansion and contraction events. The upper diagram illustrates the events observed in *E. cf. woodruffi, E. parawoodruffi* and *E. woodruffi*, while the lower diagram represents the events that occurred in *C. quadrinucleata*, nodeD, and *E. weissei*. The number within each circle denotes the count of orthologous groups

To explore the common patterns shared by CABs, we employed an unsupervised clustering approach [68], which resulted in the identification of seven distinct clusters of symbiotic bacterial profiles among 91 ciliates (Figs. 2EF and Fig. 3A). Notably, Cluster 4 was the most prevalent, accounting for 28.6% of all ciliates, followed by Cluster 2 (18.7%) and Cluster 5 (16.5%) (Fig. 2F).

To investigate the factors influencing the structure of CABs, we conducted a principal coordinate analysis (PCOA) based on the Jensen–Shannon distance of CABs [68], revealing a significant separation of seven different clusters (*P* < 0.001, Permanova test) (Fig. 3A). Among the 12 classes of ciliates, only 2 anaerobic classes (*Armophorea* and *Odontostomatea*) showed a significant enrichment in Cluster 7 (Fig. 3C, S5A). This finding corresponds to the prevalence of strictly anaerobic bacteria *Clostridia* and *Negativicutes* within this cluster [69] (Fig. 3B). In contrast, the remaining classes did not exhibit significant enrichment in any cluster (*P* > 0.05, Fisher test) (Suppl. Figure S5A), implying a strong influence of environmental factors on the symbiotic bacteria distribution.

To unveil the variations in microbial communities within unicellular ciliates in natural populations, we employed a filtering process to minimize confounding factors (see “Methods”). We focused on the most dominant bacteria, which were defined as the bacteria with the highest abundance, surpassing other taxa by at least twofold. We then assessed the consistency between 18 pairs of biological replicates from 15 ciliates that met the criteria, and consistent dominant bacterial and microbial profiles were observed in 12 pairs of biological replicates (Pearson correlation coefficient > 0.95) (Suppl. Table S4). However, the remaining six pairs displayed altered dominant bacteria, among which four pairs exhibited distinct microbial profiles (Pearson correlation coefficients < 0.1) (Suppl. Figure S5B-G) (Suppl. Table S4). In these small biological replicates, we observed that nearly one-third of the species harbored completely unique bacterial profiles, which may be due to differences in the ciliates’ ability to manage their bacterial inhabitants.

The analysis of beta diversity revealed that both the sampling site and the host (at the family level) exerted a significant influence on the structure of the symbiotic community across all ciliates (*R*^2^ = 0.84, *P* = 0.036, Permanova test). The habitat of the ciliate also showed a significant but comparatively lower effect (*R*^2^ = 0.15, *P* = 0.003, Permanova test) (Fig. 3D). Following careful consideration of co-linearity between variables (see “Methods”), we found that the sampling site emerged as the most influential factor (*R*^2^ = 0.45, *P* = 0.001, Permanova test), followed by the ciliate host (*R*^2^ = 0.30, *P* = 0.001, Permanova test) (Suppl. Figure S6A). These findings highlight the substantial role of environmental bacteria in shaping symbiotic systems with ciliates. Moreover, they align with observations from microscopic marine invertebrates, where distantly related invertebrates cohabiting the same sampling site tend to share similar symbionts [70].

To gain insights into the influence of the host on the symbiotic system, we conducted a comprehensive analysis integrating CABs and the genetic features of the host, focusing on the most diverse lineage of *Euplotes* in our cohort. We observed distinct patterns of cohabitants diversity in *Euplotes woodruffi* and *Certesia quadrinucleata*, representing two contrasting ends of the CABs pattern. Notably, in *E. woodruffi*, the bacterial community was predominantly composed of *Polynucleobacter*, which accounted for 33.3% of the sequenced data (Fig. 3E). Thus, this ciliate exhibited remarkably low alpha diversity (Fig. 3E). In contrast, CABs in *C. quadrinucl*eata exhibited the highest level of alpha diversity (Fig. 3E). The robust correlation observed among biological replicates (Pearson cor. = 0.99; *P* < 2.22e−16 for *E. woodruffi* and Pearson cor. = 0.57; *P* = 0.0006 for *C. quadrinucleata*) (Suppl. Figure S6B-C) reinforced the non-random nature of these differences. Next, we collected and analyzed the published genomes of the corresponding ciliates. By investigating the gene content related to ciliate digestive capacity, including genes associated with endocytosis, phagosome formation, lysosome function, and peroxisome activity, we found that *E. woodruffi* exhibited a higher content of these genes (Fig. 3E), indicating a more robust digestive capacity compared to *C. quadrinucleata.* Moreover, gene family analysis uncovered distinct evolutionary trajectories between *E. woodruffi* and *C. quadrinucleata*. Interestingly, *E. woodruffi* did not exhibit a contraction of gene families associated with digestive functions but displayed an expanded phagosome-related orthogroup compared to closely related species (Fig. 3F). In contrast, *C. quadrinucleata* showed a reduced scale of orthogroup expansion (Fig. 3F).

Overall, the analysis of all *Euplotes* species in the cohort unveiled a significant negative correlation between the gene content related to phagosome functions and the diversity of cohabitants (Pearson correlation coefficient = −0.71; *P* = 0.047) (Suppl. Figure S6D). This finding provides direct evidence that the survival of diversity of symbiotic bacteria is strongly influenced by the host’s digestive capacity

### Functional landscape and evolutionary patterns of CABs genes

To characterize the distribution of genes derived from CABs, we utilized Prokka [71] and Kofamscan [72] to predict and annotate genes from all bacterial-derived contigs. A total of 8,697,962 genes were identified, with 2,501,743 of them annotated to 23,730 different gene identifiers. We then categorized these genes into three major functional categories: genetic information processing (41.0%), signaling and cellular processes (40.2%), and metabolism (18.8%).

**Fig. 4:**
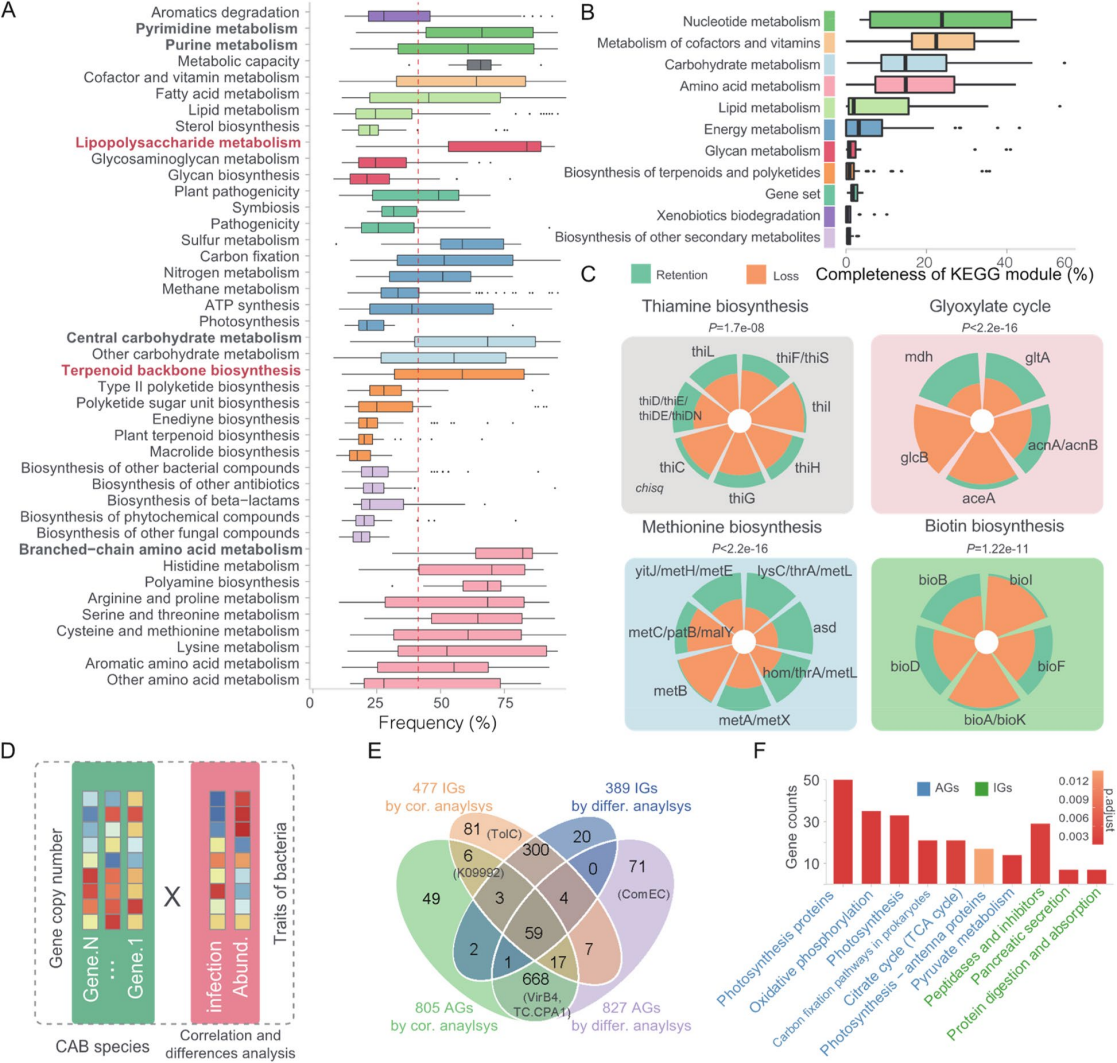
Functional distribution and loss patterns of CABs genes. **A** Frequency of CABs genes belonging to different functional modules in 91 ciliates. Each data point represents the frequency of each CABs gene in all ciliates. The line represents the mean value (41.4%) of the frequency of genes from symbiotic bacteria in ciliates. **B** The boxplot illustrates the completeness of 411 modules of the rSGB for 116 novel species. Each data point represents the integrity of each module. **C** The gene loss events within each functional module. Each pie chart represents a functional module, where each section represents the presence or absence of core genes that constitute that module. **D–F** Functional genes associated with symbiont internalization. **D** A schematic representation was used to depict the detection of functionally relevant genes. **E** A Venn diagram illustrates the overlap between infection-associated genes (IGs) and abundance-associated genes (AGs). **F** KEGG enrichment analysis was conducted for the AGs and IGs, with the color of the bars representing the *P*-value for enrichment significance

To identify key genes and pathways involved in bacteria cohabiting with ciliates, we ranked the frequency of genes present in CABs across 91 ciliate species. Subsequently, we categorized them into 411 functional modules and 11 broad functional categories based on the defined classifications in the KEGG database (Suppl. Figure S6E). We found that genes associated with amino acid metabolism, carbohydrate metabolism, metabolism of cofactors and vitamins, and nucleotide metabolism were significantly present in CABs (Fig. 4A, Suppl. Figure S6E). Similarly, when analyzing the 116 novel bacteria cohabiting with ciliates, the same modules were prominently highlighted (Fig. 4B). Notably, we observed two specific modules, namely lipopolysaccharide (LPS) metabolism and terpenoid backbone biosynthesis, which showed significantly higher gene retention compared to other modules within the same functional category (Fig. 4A). These modules may play critical roles in the intracellular life of CABs within their host. LPS has been reported to contribute to the survival of *K. pneumoniae* in amoeba phagocytosis [73].

In addition, our extensive discovery of 116 new cohabitants provided an opportunity to investigate the patterns of individual gene loss during the reductive evolution of cohabitant genomes. We observed heterogeneous rates of gene loss, even among genes belonging to the same functional modules (Fig. 4C). Notably, we identified faster loss rates (*P* < 0.001, Chi square test) for specific genes involved in fundamental functional processes. These included thiI (tRNA uracil 4-sulfurtransferase), glcB (malate synthase), metB (cystathionine gamma-synthase), and bioI (pimeloyl-ACP synthase), which are associated with thiamine biosynthesis, glyoxylate cycle, methionine biosynthesis, and biotin metabolism, respectively. Specifically, glcB serves as the unique enzyme in the glyoxylate cycle, converting glyoxylate, and acetyl-CoA into malate [74]. It has been observed that glcB is more susceptible to loss within cohabitants [75]. The malate production deficiency of symbionts may enable hosts to regulate them by controlling malate availability.

Next, we aimed to identify functional genes associated with symbiotic internalization, which can be divided into two key stages: survival (escaping or resisting phagosome digestion) and proliferation (adapting to the intracellular environment to establish a suitable niche for bacterial growth) [76]. To achieve this, we performed gene annotation on a comprehensive dataset consisting of 3,498,163 genes derived from 883 CABs species, using the eggNOG-mapper [53]. Empirically, bacteria detected in multiple ciliates exhibit enhanced survival against host phagosome digestion, while their maximum abundance reflects optimal adaptation to the host's intracellular environment. Therefore, we employed two distinct indicators, the infection rate and bacteria abundance, to assess the strength of bacterial-host interactions. We further performed differential analysis and correlation analysis to identify functional genes associated with the internalization process. Specifically, we quantified the gene copy number within CABs species and examined their correlation with the two indicators (Fig. 4D). Additionally, we evaluated whether there were significant differences in these indicators between CABs species harboring the target gene and those lacking it (Fig. 4D).

A total of 550 infection-associated genes (IGs) and 887 abundance-associated genes (AGs) were identified; 73.2% (366) of the IGs and 84.0% (745) of the AGs were supported by both differential analysis and correlation analysis (Fig. 4E). Intriguingly, only 99 genes were found to be associated with both survival and proliferation, indicating genetic independence between the two stages of internalization. KEGG enrichment analysis revealed a significant enrichment of AGs in energy metabolism pathways, including carbon fixation, oxidative phosphorylation, pyruvate metabolism, and citrate cycle. Conversely, IGs were significantly enriched in pathways related to the inhibition of digestion (Fig. 4F). Several genes within the gene set have been experimentally validated for their pivotal roles in symbiosis (Fig. 4E).

### The potential reservoirs for other symbiotic systems

To investigate the relationship between CABs and symbiotic bacteria associate with other eukaryotes, including pathogens, we examined a recent large-scale study focused on microscopic marine invertebrates, which identified 23 bacterial genera associated with marine water environments and 63 bacterial genera associated with microscopic marine invertebrates [77]. We found that a higher proportion of bacteria associated with invertebrates (53.9%) were detected in ciliates compared to environmental bacteria (13.0%). Furthermore, marine invertebrate-associated bacteria were significantly enriched in CABs (*P* = 0.0006, Fisher’s test) (Suppl. Figure S7A). Similarly, when we analyzed the overlap between 429 mammalian pathogens and CABs, we found a significant enrichment of these pathogens within CABs (*P* = 1.26e−10, Fisher’s test) (Suppl. Figure S7B). For instance, *Vibrio*, a human pathogen known for its ability to escape lysosomal digestion and survive intracellularly in human cells [78], was also found in ciliates and microscopic marine invertebrates. Additionally, we observed a significant enrichment of pathogenic bacteria within the high abundance group of CABs (*P* = 0.0065, Hypergeometric test) (Suppl. Figure S7C), suggesting the potential spillover of CABs to higher eukaryotes.

### Archaeal cohabitants of ciliates

**Fig. 5:**
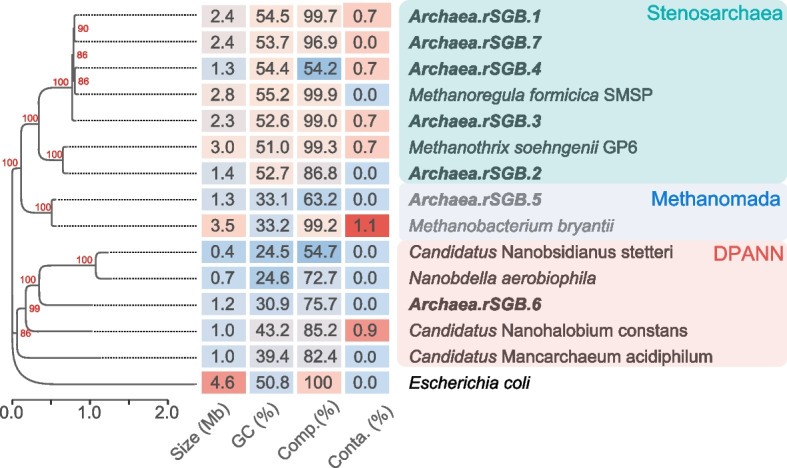
Genomic features of archaeal rSGBs. From left to right: maximum-likelihood phylogenetic tree, genome size, GC content, completeness, contamination rate, and rSGB ID. *Escherichia coli* (GCF_000005845) was used as outgroup. The scale bar represents the mean number of nucleotide substitutions per site. Bootstrap values are indicated on the tree

Archaea have been reported to establish symbiotic relationships with a wide range of organisms, including ciliates [79]. Through our analysis, we successfully assembled and binned seven archaeal genomes at the rSGB level, all of which are considered new species with an ANI of less than 83% compared to known species (Fig. 5). Among these, five belong to the *Stenosarchaea* group, one to the *Methanomada* group, and one to the DPANN group. A majority of the discovered DPANN archaea exhibit characteristics of limited metabolism, including sparse degradation and biosynthetic capabilities, and are considered to encompass a diverse range of putative cohabiting organisms [80]. Notably, Archaea.rSGB.1, found in *Brachonella* sp., and Archaea.rSGB.3, found in *Urostomides* sp., exhibit high completeness (>99%).

## Discussion

In this study, we present a comprehensive metagenome-based examination of the interactions between the entirety of the Ciliophora phylum and the prokaryotes associated with them. Our observations consistently demonstrate the occurrence of symbiotic bacteria in nearly all examined ciliate specimens, in concordance with findings in recent studies [81]. Our investigation has yielded a comprehensive repository of ciliate-associated bacteria, including 116 newly identified ciliate-associated bacteria and 7 novel ciliate-associated archaea. Additionally, we provide evidence of 883 bacteria cohabiting with ciliates. Regarding the constitution of symbionts, symbiotic associations within ciliates manifest in two distinct components: ectosymbionts and endosymbionts. Our study of CABs revealed their potential resistance to host food vacuole digestion, implying that a significant portion of the CABs may function as endosymbionts. However, it is imperative not to perceive ectosymbionts as arbitrary attachments. The prevalence of ectosymbionts is extensively documented across a diverse spectrum of ciliate species [2, 28, 82, 83], highlighting their widespread occurrence in ciliate communities. Ectosymbionts, in turn, exhibit evolutionary adaptations to their respective hosts. An illustrative example is the longitudinal division of an ectosymbiont observed on the surface of the ciliate *Kentrophoros* [82], which can facilitate the maintenance of attachment for daughter cells and prevent detachment from the host surface.

Of CABs, 85% was detected only at the contig level, indicating that they have a low biomass and suggesting a possible role as transient symbionts. Nevertheless, these CABs present the potential to survive the digestive process within ciliate phagosomes. Given the conserved maturation-dependent pathways of phagosomes, it is improbable that most CABs possess host-specific anti-digestive strategies, as evidenced by the identification of CABs with multiple eukaryotic hosts. For instance, we detected *Polynucleobacter* in 15 distinct ciliate species. It is important to note that most of them were only assembled into contigs. A high-quality genome assembly was achieved solely in Euplotes, emphasizing the need for additional studies in the future. In addition, some of the bacteria previously found in amoebae (e.g., *Legionella pneumophila* [49]*,* “*Candidatus* Finniella inopinata” [66], and *Microcystis aeruginosa* [67]) were also found in ciliates in our and other studies [44, 46]. Furthermore, we have identified a significant overlap between these contig-level CABs and symbionts of marine invertebrates, as well as animal pathogens. The potential diversity of hosts for symbiotic bacteria suggests that the low biomass observed may be due to the inadequacy of our sampling, resulting in their optimal hosts not being exposed. Consequently, symbiotic bacteria with low biomass remain ecologically and evolutionarily significant.

We have also observed that even individuals of the same ciliate species residing in the same location exhibit significant variations in their cohabitant profiles. This observed variation in cohabitant composition among individual ciliates aligns with findings in previous studies [46, 84]. Notably, this phenomenon extends beyond the confines of the *Polynucleobacter*-*Euplotes* symbiotic system [85], suggesting that the susceptibility to symbiotic bacteria replacement is not exclusive to this specific system. Symbiotic bacteria may be replaced by other bacteria strains from the surrounding environment [85], and in certain instances, even by bacteria belonging to a different taxonomic class [85]. This dynamic process, characterized by the continuous internalization and replacement of symbiotic bacteria, substantially contributes to the overall diversity observed among ciliate symbionts, emphasizing the importance of studying the internalization process of symbiotic bacteria. The expansive host range of some CABs implies an active role for bacteria in establishing and maintaining the symbiotic system.

In our study, we made significant discoveries regarding the genetic basis of symbiotic internalization, specifically the processes of survival and proliferation, which appear to be genetically independent. We identified a total of 550 and 887 genes significantly associated with these two stages, providing a comprehensive gene set that sheds new light on the molecular mechanisms underlying symbiotic processes. Among the identified genes, several have been previously characterized as playing crucial roles in endosymbiosis (Fig. 4E). For instance, TolC, which enables *Legionella pneumophila* to persist within *Paramecium tetraurelia* cells by inhibiting phagosome formation [30], and the gene Ech_0230, which facilitates the growth of the intracellular pathogen *Ehrlichia chaffeensis* in both reservoir hosts (deer) and incidental hosts (dog), are noteworthy examples [32]. Our gene set also encompasses other genes associated with bacterial symbiosis, including the trehalose-utilizing gene (K09992) associated with osmotic stress tolerance [33], the exogenous DNA transformation-associated gene ComEC [34], and the T4SS-associated gene VirB4 [35]. These findings expand our understanding of the genetic factors involved in bacterial symbiosis and provide valuable insights into the molecular processes underlying these complex interactions.

In summary, this study expands our knowledge by presenting a comprehensive collection of prokaryotes living in cohabitation with ciliates. It elucidates the prevalence of symbionts derived from free-living bacteria and provides insights into the genetic mechanisms underlying internalization. These findings deepen our understanding of the complex interactions between ciliates and their cohabitants and establish a foundation for future investigations focused on unraveling the intricate mechanisms involved in the establishment, maintenance, and evolutionary implications of eukaryotic-bacterial symbiotic systems.

## Methods and materials

### Specimen collection and metagenome sequencing

The ciliate specimens were collected from seawater, freshwater, and sediment in Qingdao, China. Ciliates were gathered as a population, encompassing a range of 10 to 20 individuals. The specimens were placed in Petri dishes and ciliate cells were isolated using glass pipettes. Subsequently, these individuals underwent a preliminary classification under a microscope, based on their live morphological features such as body size, cellular shape, motility patterns, cortical granules, adoral zone features, number of contractile vacuoles, and somatic kineties. Next, we conducted a secondary confirmation by randomly selecting three to five cells from the initial screening for protein silver staining. This step was conducted by skilled specialists with expertise in ciliate taxonomy. Established keys and reference guides were consulted during this morphological analysis. In cases where ciliate identification based solely on morphology posed challenges, 18S sequencing was employed to ensure accurate taxonomic assignment. If morphological assessments confirmed that silver-stained individuals belonged to the same species, we deemed the collected individuals taxonomically consistent and representative of the same species. Only individuals subjected to this rigorous validation process were deemed eligible for the subsequent sequencing step.

To ensure the vitality of the cells before silver staining and sequencing, a cultivation period was employed, typically not exceeding 7 days. The *Escherichia coli* K-12 and a non-pathogenic strain of *Klebsiella pneumoniae* were utilized as food. During the cultivation period, some cells may undergo division. Consequently, our sequencing efforts were targeted specifically at cells displaying normal size and morphology, with preference given to those that had not undergone division.

Prior to DNA extraction, the ciliate cells were subjected to three to five washes with phosphate-buffered saline buffer (without Mg^2+^ or Ca^2+^) to remove any surface contaminants [86]. Following the washes, a starvation period of 12 to 48 h was implemented to exhaust undigested bacteria [87]. To ensure a comprehensive retrieval of bacteria, a pooling strategy was adopted, where 1~3 cells were combined as a sample, with an average of 2.73 biological replicates per species; 76% of the samples contained only one cell.

To mitigate the potential impact of preferential amplification methods on bacterial identification, for the amplification of genomic DNA, two widely-used methods, namely multiple displacement amplification (MDA) and multiple annealing and looping based amplification cycles (MALBAC) [36], were randomly applied in the biological replicates for each ciliate. The REPLI-g Single Cell Kit (Qiagen) based on MDA technology, or the Single-Cell WGA Kit (Yikon, YK001A) based on MALBAC technology were used for genomic DNA amplification, following the manufacturer’s guidelines. Subsequently, sequencing libraries were constructed using the TruSeq Nano DNA HT Sample Preparation Kit (Illumina). High-throughput sequencing was performed on the Illumina NovaSeq 6000 platform using PE150 sequencing chemistry. In total, we generated 246 metagenomic sequencing datasets for 91 species from 81 genera.

### Metagenome assembly, binning, and annotation

We use TrimGalore (https://github.com/FelixKrueger/TrimGalore) for quality control and trim reads. To remove any potential host contamination in raw sequencing reads, a two-step filtration strategy was employed. First, contig-level decontamination was performed. As the ciliate macronuclear genome is typically no larger than 100 M, a subset of 10 million reads from the specimen sequencing data was utilized by Megahit (version 1.1.3) to expedite assembly [37]. After taxonomy classification by Kraken2 [88] with contigs greater than 1 kb, the reads origin from eukaryotic contigs were removed. Next, read-level decontamination was performed by Kraken2 with default parameters based on the RefSeq non-redundant proteins database, and all eukaryotic reads were subsequently filtered out. A total of 47% of reads were removed through the two steps. After filtering, the reads were reassembled using Megahit, producing a total of 33,494,587 contigs. By using MMseqs2 [38] with parameters of “lca-mode 3 orf-filter 0” based on the database of UniRef90, a total of 6,042,995 bacterial contigs were identified, which were then binned by Metabat2 (version 2.12.1) [89] resulting in the identification of 265 genomic bins that met the criteria of over 50% genome completeness and less than 5% contamination. Assembling and binning were performed for each sample. To confirm the accuracy of contig classification, taxonomic classification was also carried out using Kaiju [39] with default parameters based on the RefSeq non-redundant proteins database. To assess the magnitude of unknown bacteria within ciliates, ten healthy adult fecal metagenomic sequence data were downloaded [90] and subsequently assembled and annotated using the same procedures used to detect CABs.

Next, we employed Prokka [71] (version 1.14.6) for gene prediction of all bacterial-derived contigs, and Kofamscan (version 1.3.0) [72] was utilized to annotate their functions with the parameter “-E 0.01”. Subsequently, the gene was assigned the KO identifier with the minimum e-value. A total of 8,697,962 genes were identified, with 2,501,743 of them annotated to 23,730 different gene identifiers.

BAT was utilized to classify the taxonomic composition of the bins [91]. This tool entails mapping predicted ORFs against a protein database and employs a voting-based classification of the entire MAG based on the classification of individual ORFs. For the genus analysis of *Legionella* and *Polynucleobacter*, we employed FastANI (version 1.32) [92] to identify the closest phylogenetic relatives of the bin. In accordance with recommendations from the reference [93], organisms with an ANI greater than 74% were considered potential members of the same genus. Finally, all related bins underwent confirmation through the construction of phylogenetic trees, incorporating known species of the genus. The coverage profile of contigs was determined using Salmon (version 0.10.1) [94].

### Representative species-level genomic bin (rSGB) and novelty assessment

To demultiplex bins, bins were selected using thresholds of completeness >50% and contamination <5% [95]. CheckM (https://github.com/Ecogenomics/CheckM) [96] was used to assess the N50, completeness, and contamination. Next, FastANI (version 1.32) [92] was used to calculate the average nucleotide identity (ANI) distance among all bins. Based on ANIs over than 95%, bins were classified into species-level clusters. From each cluster, we selected a representative single genomic bin (rSGB) based on maximum completeness and minimum contamination. In total, we obtained 148 bacterial rSGBs. 15,495 genomes were downloaded from NCBI Reference Sequence Database as reference genomes and ANI distances to rSGBs were calculated using FastANI. As previously defined [40], an rSGB with a maximum ANI >95% was regarded as known species, the value <83% as novel species, and the value in between as intermediate.

### Functional annotation of ciliate-associated bacteria

The 546 genera and 883 species of CABs were determined using a threshold of >100 kb total length. To identify functional genes associated with the abundance and frequency of CABs in their hosts, protein sequences of species were downloaded from the NCBI Reference Sequence Database. eggNOG-mapper [53] was used to annotate 3,498,163 genes with parameter *--tax_scope bacteria*, assigning each gene to a KO identifier. Then, the rank sum test was used to compare the differences in traits (abundance and frequency) between bacteria containing this KO identifier and those not containing it. Spearman correlation analysis was performed using the *function cor.test(method='spearman')* in R to assess the correlation between KO identifier copy number and traits. We used *P* < 0.01 as the significance threshold and identified a total of 550 infection-associated genes and 887 abundance-associated genes.

The phenotypes were predicted by PhenDB [58], which uses a machine learning approach to predict bacterial phenotypes based on genomic features, and MacSyFinder [59], which searches for specific protein families and genomic regions involved in bacterial phenotypes using a hidden Markov model-based approach, with default parameters.

KEGG enrichment analysis was performed using the function enricher in the R package “clusterProfiler” (https://github.com/YuLab-SMU/clusterProfiler).

### Module annotation of genes

After gene KO annotation of all bacterial-derived contigs within each ciliate and the genome of each rSGB. Information on the relationship between gene and module correspondence was obtained from the R package KEGGREST (https://bioconductor.org/packages/KEGGREST/). Genes with the same KO identifier are defined as the same gene. We then calculated the occurrence frequency of each gene in the bacterial-derived contigs among 91 ciliates. Completeness analysis of the pathway modules in bacteria was performed by enrichM (https://github.com/geronimp/enrichM).

### Clustering of CABs abundance profiles

The abundance of CABs in each sample at the class level was tabulated and the abundance of ciliates was used as a mean abundance of samples. Following the published method [68], the abundance profiles underwent unsupervised clustering using the Jensen–Shannon distance and partitioning around medoid algorithm. To determine the optimal number of clusters that best captured the underlying structure in the data, we employed the Calinski–Harabasz (CH) index. The CH index evaluates the quality of clustering results by considering the between-cluster dispersion and the within-cluster dispersion. Higher CH index values indicate better-defined clusters. To determine the optimal number of clusters, we iterated through 1 to 20 clusters, and the optimal CH index was found to be cluster number equal to 7.

Linear discriminant analysis effect size analysis (LEfSe) was performed using the function *lefser* of R package lefser (https://github.com/waldronlab/lefser). The linear discriminant analysis (LDA) scores represent the effect size of each abundant class. Signature class was detected with threshold LDA score greater than 3.

### Diversity analysis of CABs

The microbial table was normalized to by total coverage using the function of *transform(transform = “compositional”)* in the R package microbiome (version 1.6.0) (https://github.com/microbiome/microbiome). The Shannon index and observed genus number (richness index) were calculated by function diversity in the R package vegan (version 2.6.2) (https://github.com/vegandevs/vegan). Statistical significance was calculated using two-tailed Wilcoxon test in R.

### Removal of co-linearity of variables

To assess the factors affecting ciliate symbionts, we filtered the specimens to exclude confounding factors based on the following criteria: firstly, we screened the sampling sites where multiple species of ciliates were collected. Secondly, there should be at least two biological replicates of each ciliate, and finally, each ciliate should contain both MDA-amplified and MALBAC-amplified samples. Our analysis was restricted to specimens subjected to the same amplification method (MDA or MALBAC). A total of 69 samples were used for follow-up analysis.

Next, 37 MALBAC-treated samples of 18 species from eight sampling sites were used for subsequent analyses (Suppl. Table S4), as MALBAC-treated samples exhibited higher alpha diversity. T-distributed stochastic neighbor embedding (tSNE) analysis was used to visualize the similarity of the symbionts by R package Rtsne (version 0.16) (https://github.com/jkrijthe/Rtsne). Permutational multivariate analysis of variance (PERMANOVA) was used to test the difference between groups of traits with the function of *adonis2(method=“bray”)* in the R package vegan.

### Phylogenetic analysis of ciliate and bacterial cohabitants

The cladogram of ciliates was manually constructed, referencing the studies by Gao et al. [97] and Fernandes et al. [98]. For the analysis of the bacterial cohabitants such as *Legionella*, *Polynucleobacter*, and *Syntrophorhabdia*, we downloaded the genomes of these taxa from the NCBI database and performed phylogenetic analyses along with the assembled genomes. First, genomes were processed using checkM [96] to automatically extract, concatenate, and align proteins from a set of 43 phylogenetically informative marker genes including ribosomal proteins and RNA polymerase domains. This process involves Prodigal [99] and HMMER [100] for marker gene identification and extraction, followed by MAFFT [101] to perform multiple sequence alignments. Subsequently, phylogeny reconstruction was carried out using RAxML [102] with the PROTGAMMAILGF model. A combined outgroup consisting of *Escherichia coli* (GCF_000005845), *Lactococcus chungangensis* (GCF_002441885), and *Geobacter sulfurreducens* (GCF_000007985) were used to root the tree.

For the 148 bacterial rSGBs, FastTree (version 2.1) [103] was used to construct the phylogenetic tree after processing 43 markers using the same method due to the high number of rSGBs. The trees of *Legionella*, *Polynucleobacter*, *Syntrophorhabdia*, and rSGBs are available on Github (https://github.com/bioinfo-biols/Supplementary_materials_CiliateSymbiont_2024) in Newick format. Features of the symbiotic genomes were analyzed and visualized using Prolsee (https://proksee.ca/) [104].

### Gene family analysis

The genome and protein sequences of eight *Euplotes* species were obtained from our previous study [105]. *Oxytricha trifallax* (http://oxy.ciliate.org/index.php/home/downloads) was used as an outgroup in the phylogenetic analysis. OrthoFinder (version 2.3.12) [106] was used to find homologs among them. DupliPHY [107] was used to determine gene families with expansions, contractions, gain, and/or loss among Euplotes. Briefly, DupliPHY was employed to reconstruct the presence and size of gene families in the most recent common ancestor of *E. woodruffi* and *C. quadrinucleata*, as well as earlier common ancestors. The comparison of gene numbers between *E. woodruffi* or *C. quadrinucleata* and their ancestral node shared with related ciliates was performed.

All protein-coding genes in eight Euplotes were annotated for gene function using Kofamscan (version 1.3.0) [72] with the parameter “-E 0.01”. And then the KO identifier with the minimum e-value was assigned to this gene.

### Relationship of CABs with other eukaryotes

The bacteria associated with microscopic marine invertebrates were downloaded from a recent study [70] . Bacteria enriched in samples collected in water column were included in this analysis. In total, there were 23 genera associated with marine water environment and 63 genera associated with microscopic marine invertebrates. The 429 mammalian pathogens at the genus level were downloaded from Enhanced Infectious Diseases Database [108].

### Assembly and phylogenetic analysis of archaeal cohabitants

We analyzed archaeal cohabitants using the same procedures and parameters as for the bacterial cohabitant analyses. In phylogenetic analysis, *Escherichia coli* (GCF_000005845) was used as the outgroup. The published archaeal genomes, including *Methanobacterium bryantii* (GCF_002287175) from the Methanomada group, *Methanothrix soehngenii* GP6 (GCF_000204415) and *Methanoregula formicica* SMSP (GCF_000327485) from the *Stenosarchaea* group, and *Nanobdella aerobiophila* (GCF_023169545), “*Candidatus* Nanohalobium constans” (GCF_009617975), “*Candidatus* Mancarchaeum acidiphilum” (GCF_002214165), and “*Candidatus* Nanobsidianus stetteri” (GCF_003086415) from the DPANN group, were included in the phylogenetic analysis. The tree is available on Gtihub (https://github.com/bioinfo-biols/Supplementary_materials_CiliateSymbiont_2024).

### Supplementary Information

**Additional file 1.** Supplementary data

## Data Availability

The raw sequence data and assemblies of the representative single genomic bins in this study have been deposited in National Genomics Data Center under the accession code PRJCA019347 (https://ngdc.cncb.ac.cn/bioproject/browse/PRJCA019347).
