# Supplementary material for: Exploring the landscape of symbiotic diversity and distribution in unicellular ciliated protists
Source: Microbiome. 2024 May 24;12:96. doi: 10.1186/s40168-024-01809-w (PMC11127453; doi:10.1186/s40168-024-01809-w)
Supplement: Supplementary file 2 — Additional file 1. Supplementary data [file 40168_2024_1809_MOESM1_ESM.pdf]

Supplementary Materials for

Exploring the landscape of symbiotic diversity and distribution in unicellular ciliated protists

Bing Zhang[1,2,^], Liwen Xiao[2^], Liping Lyu[3], Fangqing Zhao[1,2,4,*], Miao Miao[1,2,*]

This PDF file includes:
Figs. S1 to S7
Tables S1 to S4

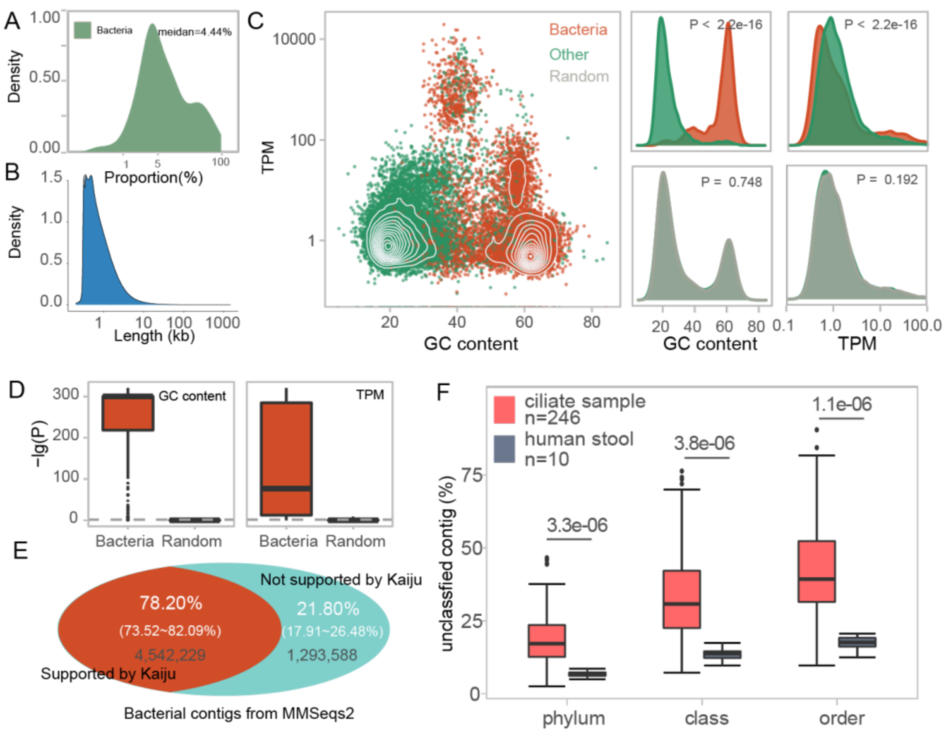

Figure S1. Metagenome assembly and identification of ciliate-associated bacteria. (A) The length distribution of bacterial contigs. A total of 6,042,995 ciliate-associated bacterial contigs were analyzed. The mean contig length was 1197.44 bp. (B) Distribution of bacterial content among specimens. The bacterial-derived reads accounted for 4.44% of the total sequencing data. The density was generated automatically using the "geom_density" function in ggplot. A transcript per million (TPM) like calculation to normalize the length of the contig and the sequencing depth of the sample when assessing the coverage of the contig. (C-E) Accuracy evaluation of contig classification. (C) The distribution of TPM and GC content between bacterial and host contigs. (D) Comparison of GC content (left) and TPM (right) between bacterial contigs and randomly selected control contigs for all specimens. The dashed line indicates the *P* value of 0.05. (E) The accuracy of contig taxonomic classification evaluated by another software. Numbers in brackets are 95% confidence intervals for the means. (F) Comparison of unclassified contigs in ciliate samples and human stool samples. The median of unclassified contigs assembled from ciliate samples were 17.18%, 30.77% and 39.26% at the phylum, class and order levels, respectively, compared to 6.66%, 13.78% and 17.60% for the human stool samples. Box-plot elements are defined as: center line, median; box limits, upper and lower quartiles; whiskers, 1.5× interquartile range; points, outliers. *P* values were measured by two-tailed Wilcoxon test. n, number of specimens. The proportion was calculated by dividing the total length of unclassified contigs by the total length of all contigs, and contigs smaller than 1 kb were filtered out.

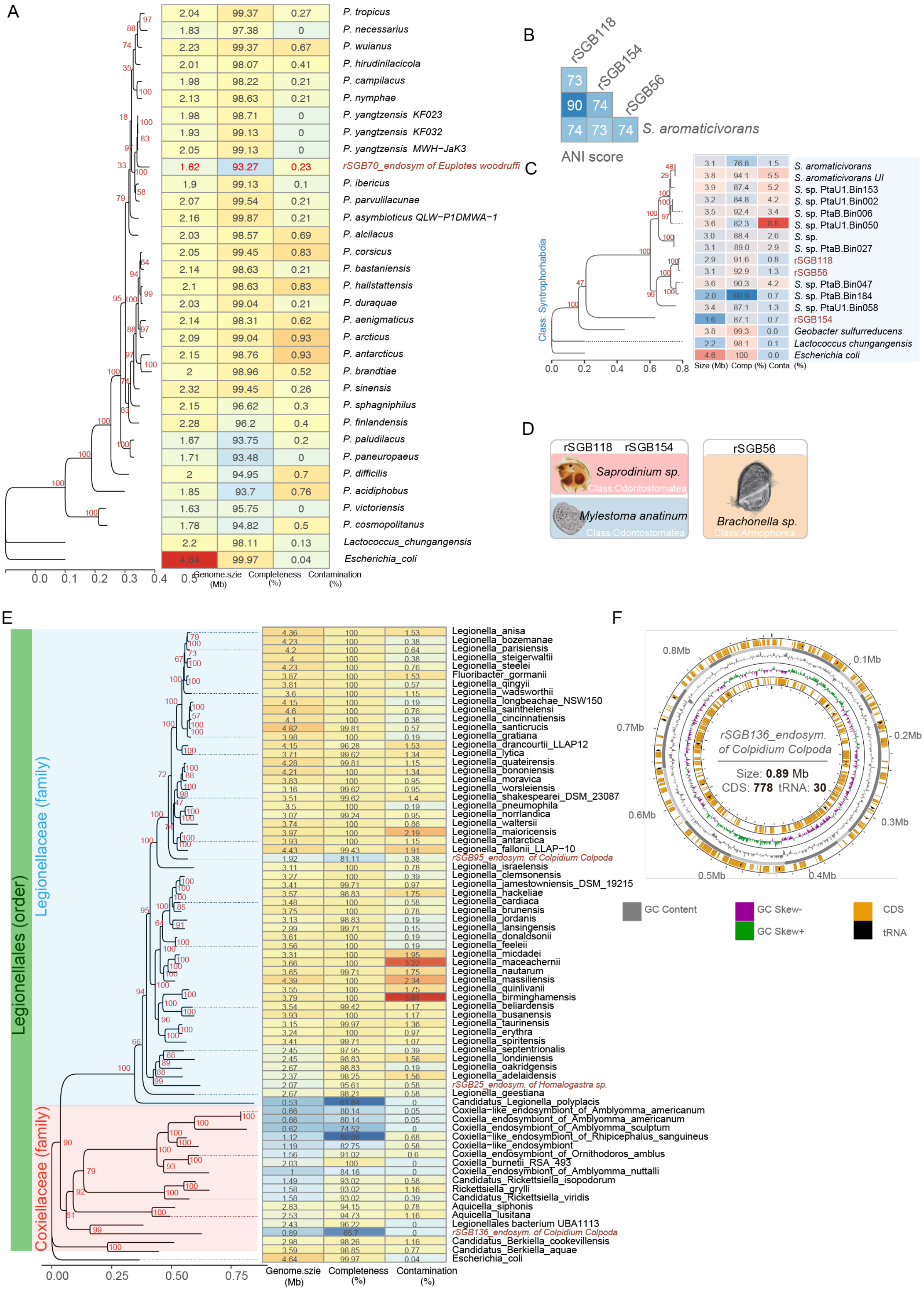

Figure S2. New symbionts in ciliates. (A) An approximately maximum-likelihood phylogenetic tree of *Syntrophorhabdia* was constructed. (B) An approximately maximum-likelihood phylogenetic tree of *Syntrophorhabdia* was constructed. From left to right: Maximum-likelihood phylogenetic tree, genome size, GC content, completeness, contamination rate, and rSGB ID. The *Escherichia coli* (GCF_000005845) was used as outgroup. The scale bar represents the mean number of

nucleotide substitutions per site. Bootstrap values are indicated on the tree. (C) Average nucleotide identity assessment was performed to compare three symbionts (rSGB118, rSGB154, and rSGB56) with the representative species *Syntrophorhabdus aromaticivorans*. (D) The host species of the three symbionts. (E) The approximately maximum-likelihood phylogenetic tree of the order Legionellales. (F) Genomic characteristics of arepresentative endosymbiont, namely rSGB136. From the outer ring to the inner ring arethe genes of positive strand. GC content. GC skew and negative strand genes. respectively.

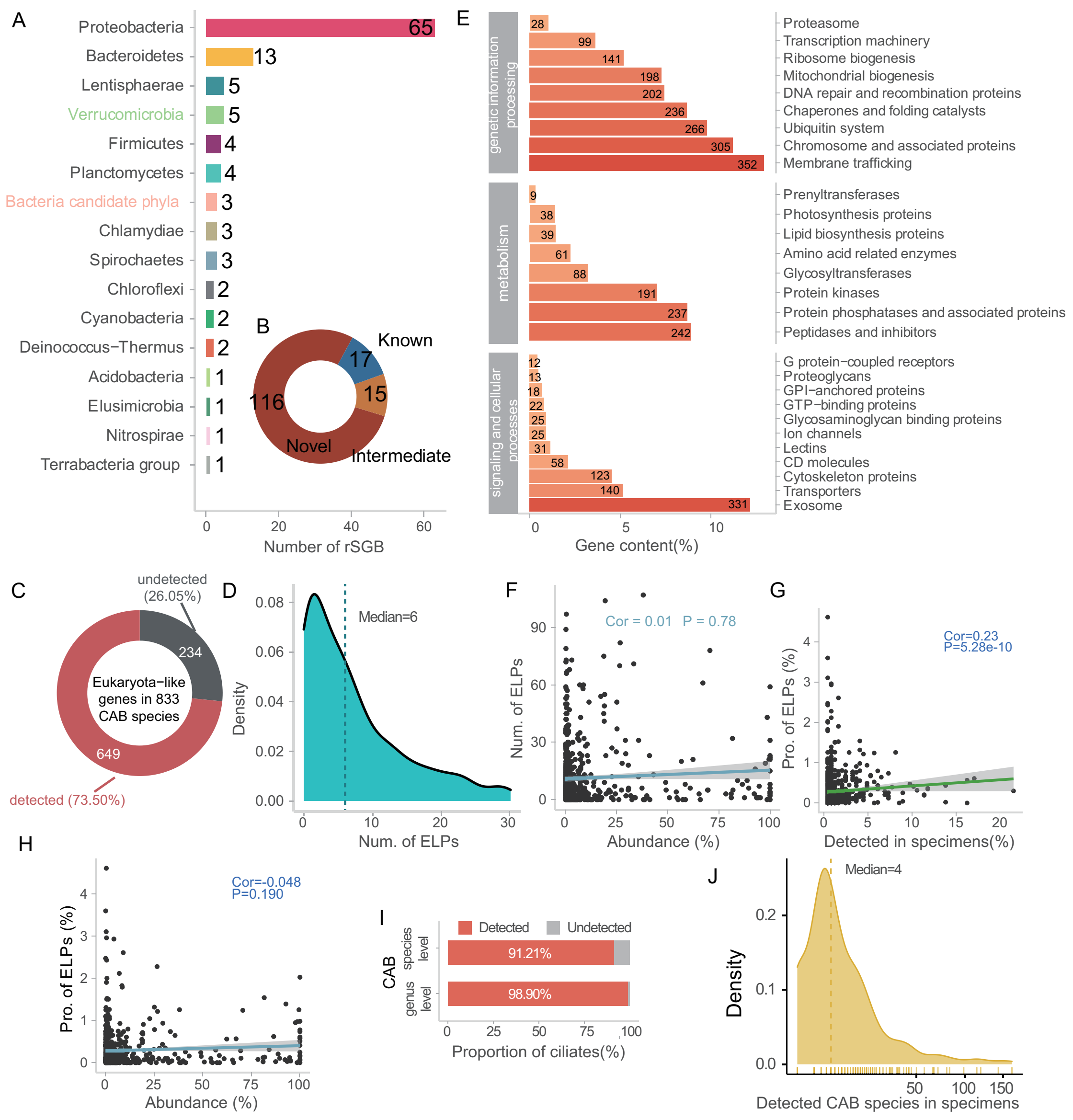

Figure S3. Characteristics of ciliate-associated bacteria. (A-B) The taxonomy of representative single genomic bins. (A) The count of novel rSGB in each phylum or clade. (B) The percentage of novel bacterial rSGBs. (C-H) Relationship between eukaryotic like proteins (ELPs) and symbionts. (C) ELPs were identified in 73.5% of CABs. (D) The distribution of the ELP counts in CAB species. The mean and median of the ELPs were 11.3 and 6, respectively. (E) KEGG functional classification of the ELPs. The color of bars represents the proportion of total ELPs. (F) Spearman correlation between the number of ELPs in CABs and their abundance in the host. (G-H) Spearman correlation between the proportion of ELPs in CAB and their prevalence (G) and abundance (H) in the host. (I) Detection of CABs in ciliates at species and genus level. (J) Distribution of 883 CAB species among 246 specimens. Each short bar denotes one specimen.

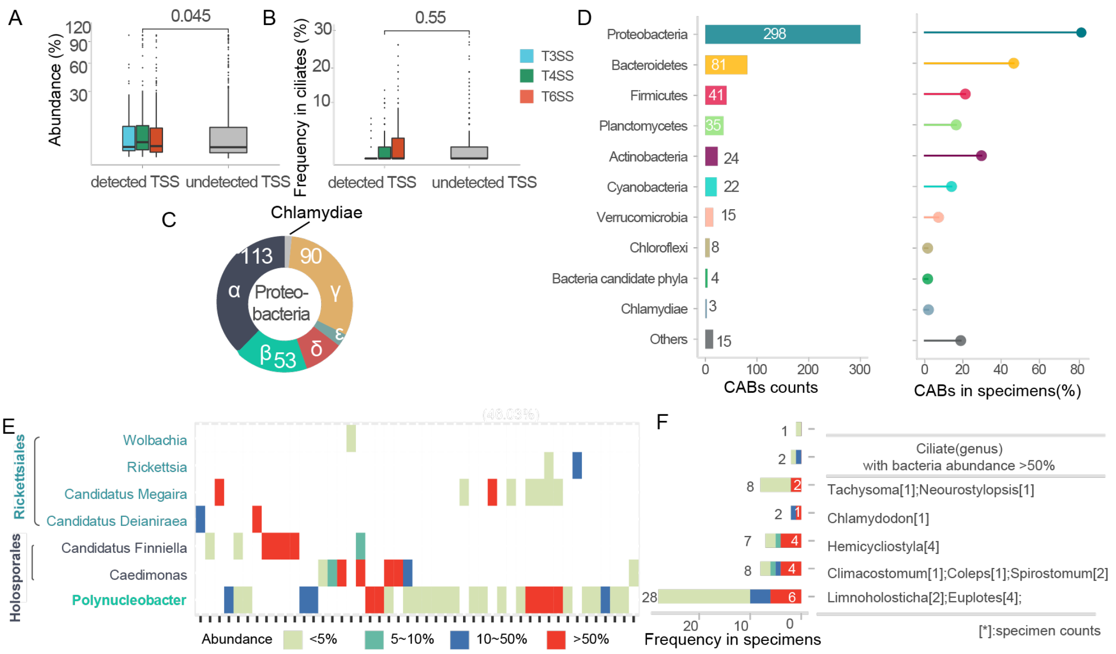

Figure S4. **Insights into CABs: infection rate, abundance, symbiotic distribution, and taxonomic analysis.** (A-B) Comparison of infection rate (A) and abundance (B) between CABs with and without secretion systems. The statistical significance was assessed using a two-tailed Wilcoxon test. (C) Distribution of *Proteobacteria* phylum at the class level. (E) Taxonomy (right) and frequency (left) of the 546 CAB genera at the phylum level. (F) Abundance of representative symbiotic bacteria in hosts. Each color block represents a sample. For instance, the *Polynucleobacter* detected in a total of 28 samples, originating from 15 ciliate species. These include one *Euplotes* ciliate and 14 non-Euplotes ciliates. Specifically, as follows: *Euplotes woodruffi* (n=4), *Brachonella* sp. (n=3), *Urocentrum* sp. (n=3), *Vorticella* sp. (n=3), *Campanella umbellaria* (n=2), *Epistylis* sp. (n=2), *Limnoholosticha viridis* nov. sp. (n=2), *Spirostomum* sp. (n=2), *Apodileptus visscheri* (n=1), *Caenomorpha medusula* (n=1), *Hemiamphisiella* sp. (n=1), *Licnophora lyngbycola* (n=1), *Metopus* sp. (n=1), *Strobilidium gyrans* (n=1), *Vaginicola* sp. (n=1). Here, "n" represents the number of samples detected *Polynucleobacter*. Different colors represent different abundance levels. (G) Statistics on the frequency of representative symbiotic bacteria present in the specimens. The number at the top of the bar represents the frequency of taxa detected in the specimen. The numbers in the red bar indicate the count of samples where the abundance exceeds 50%. The table on the right show hosts with >50% abundance.

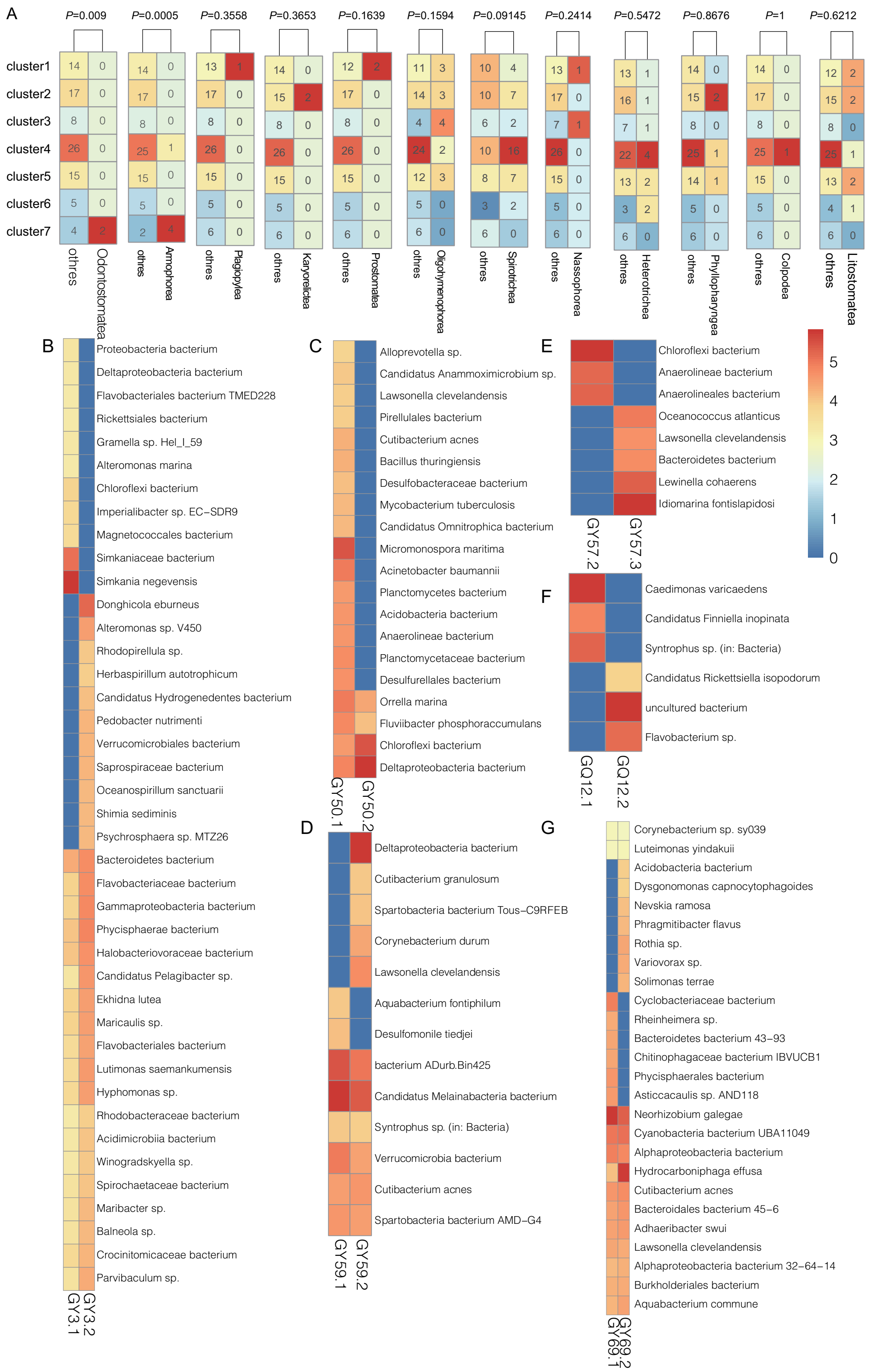

Figure S5. (A) Enrichment analysis of different CAB patterns at the host's class level. P-values were calculated using Fisher's test. (B-G) Variation in the bacterial community at the individual level of ciliates. The rows of the heatmap represent the two biological replicates of the host, the columns represent different symbiotic bacteria, and the colors indicate the abundance of the symbiotic bacteria.

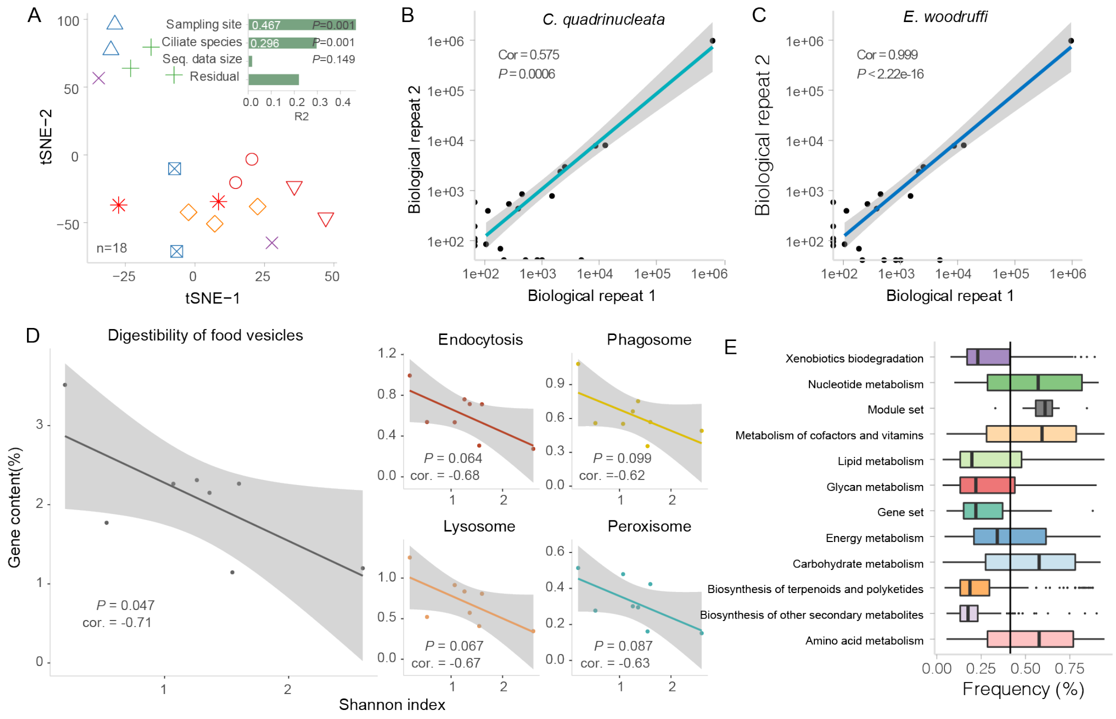

Figure S6. Factors influencing the microbiota structure and functional analysis. (A) t-SNE analysis was conducted to examine the influencing factors of the ciliate symbiotic community. The R^2 value and P-value were calculated using the Permanova test. Each dot on the plot represents a distinct ciliate species, with the same color and shape indicating samples from the same sampling site. (B-C) Spearman correlation analysis was performed to assess the relationship between different biological replicates. The left panel (B) represents the analysis for *Certesia quadrinucleata,* while the right panel (C) represents the analysis for *Euplotes woodruffi*. (D) Pearson correlation analysis was employed to examine the correlation between gene content and the Shannon index of intracellular symbionts. The digestibility of food vesicles was defined by the inclusion of genes associated with endocytosis, phagosome, lysosome, and peroxisome. (E) The frequency of CAB genes from different functional classes was examined across 91 ciliate species.

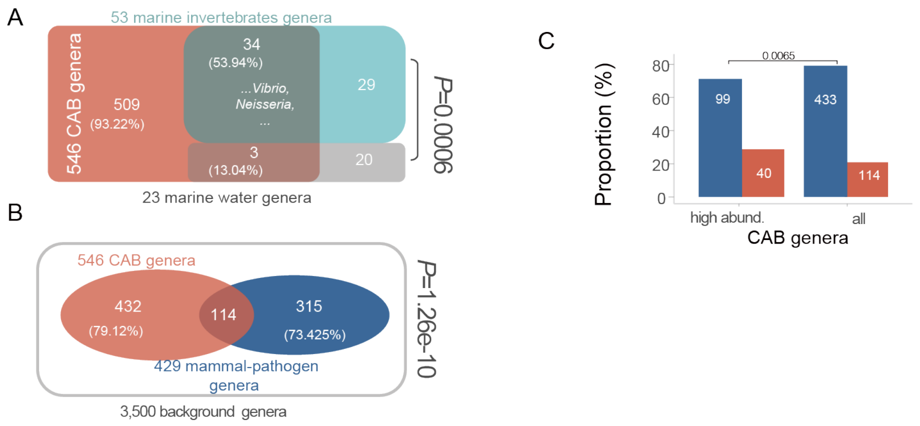

Figure S7. The potential reservoirs for other symbiotic systems (A) The Venn diagram illustrates the overlap between CABs and bacteria associated with marine invertebrates. (B) The Venn diagram represents the overlap between CABs and mammal pathogens. Fisher's test was used for statistical analysis in panels E and F. (C) High abundance of CAB is significantly enriched in mammalian pathogens. The significance was determined using the hypergeometric test. The blue bar represents CAB, while the orange bar represents mammal pathogens. Genera with a maximum abundance greater than 10% in the specimen were defined as high-abundance genera.

Table S1. Specimen information of the collected ciliate species.

SpeciesName	SampleID	SpeciesIdentifier	Amplification	SequenceReads	AfterFilterReads	BacterialContent	Genus_shannon	Genus_simpson	Genus_richness	CellNumber
**Plagiopyla nasuta**	GQ1.2	SpeciesID:1	MDA	73910316	49244383	0.04	1.90	0.51	2.00	1
**Plagiopyla nasuta**	GY1.1	SpeciesID:1	MALBAC	70759176	53367375	0.04	0.24	0.07	2.00	1
**Loxodes**	GQ10.2	SpeciesID:10	MDA	94656435	63250237	0.02	2.42	0.59	3.00	1
**Loxodes**	GY10.1	SpeciesID:10	MALBAC	65278788	49791888	0.21	1.89	0.62	13.00	1
**Loxodes**	GY10.2	SpeciesID:10	MALBAC	80919834	61308149	0.24	1.62	0.58	11.00	1
**Trachelocercidae sp.**	GY11.1	SpeciesID:11	MALBAC	68688926	37528158	0.11	1.98	0.71	7.00	1
**Trachelocercidae sp.**	GY11.2	SpeciesID:11	MALBAC	73996655	51163810	0.23	1.70	0.56	9.00	1
**Coleps**	GQ12.1	SpeciesID:12	MDA	68817049	41575572	0.08	1.69	0.48	3.00	1
**Coleps**	GQ12.2	SpeciesID:12	MDA	78308041	43065956	0.03	1.80	0.46	3.00	1
**Coleps**	GY12.1	SpeciesID:12	MALBAC	74559529	52892359	0.20	1.51	0.37	11.00	1
**Coleps**	GY12.2	SpeciesID:12	MALBAC	90480533	60733562	0.05	1.83	0.46	15.00	1
**Vaginicola**	GY13.1	SpeciesID:13	MALBAC	107732548	83494547	0.33	1.88	0.47	67.00	1
**Vaginicola**	GY13.2	SpeciesID:13	MALBAC	103439392	74993065	0.23	1.84	0.46	39.00	1
**Thuricola**	GY14.1	SpeciesID:14	MALBAC	87209123	52006590	0.09	1.94	0.50	28.00	1
**Thuricola**	GY14.2	SpeciesID:14	MALBAC	92141259	56196171	0.09	1.83	0.45	29.00	1
**Kiitricha marina**	GQ15.2	SpeciesID:15	MDA	73414392	25694182	0.03	2.88	0.65	0.00	1
**Kiitricha marina**	GY15.1	SpeciesID:15	MALBAC	88812124	59011185	0.05	2.70	0.68	12.00	1
**Kiitricha marina**	GQ15.1	SpeciesID:15	MDA	74083375	27430361	0.03	2.90	0.67	1.00	1
**Pseudoamphisiella alveolata**	GQ16.3	SpeciesID:16	MDA	66768262	28979607	0.06	1.65	0.46	2.00	>1
**Pseudoamphisiella alveolata**	GY16.1	SpeciesID:16	MALBAC	95882348	63582157	0.02	2.47	0.61	2.00	1
**Pseudoamphisiella alveolata**	GY16.3	SpeciesID:16	MALBAC	79030794	53026077	0.01	2.46	0.63	2.00	>1
**Uronychia setigera**	GQ17.2	SpeciesID:17	MDA	88688644	50218923	0.02	1.45	0.51	1.00	1
**Uronychia setigera**	GQ17.3	SpeciesID:17	MDA	82815384	47359851	0.02	2.19	0.66	1.00	>1

**Uronychia setigera**	GY17.1	SpeciesID:17	MALBAC	68660965	44745515	0.02	1.87	0.53	1.00		1
**Uronychia setigera**	GY17.2	SpeciesID:17	MALBAC	73811811	47096219	0.02	1.98	0.54	1.00		1
**Pleuronema sp.**	GY18.2	SpeciesID:18	MALBAC	114052613	75371000	0.04	2.03	0.62	4.00		1
**Pleuronema sp.**	GY18.4	SpeciesID:18	MALBAC	78076501	51090414	0.08	2.08	0.61	11.00	>1	
**Pleuronema sp.**	GY18.5	SpeciesID:18	MALBAC	78290999	49908820	0.06	1.99	0.57	11.00	>1	
**Epistylis**	GY19.2	SpeciesID:19	MALBAC	100298768	64871890	0.08	2.49	0.68	23.00		1
**Epistylis**	GY19.3	SpeciesID:19	MALBAC	95114592	63667560	0.09	2.27	0.66	17.00	>1	
**Strombidinopsis sp**	GQ2.2	SpeciesID:2	MDA	85412150	72265792	0.26	2.29	0.59	37.00		1
**Strombidinopsis sp**	GY2.1	SpeciesID:2	MALBAC	71992178	45720211	0.11	1.45	0.60	11.00		1
**Strombidinopsis sp**	GY2.2	SpeciesID:2	MALBAC	70479978	41005216	0.04	2.40	0.61	23.00		1
**Pseudokeronopsis cf. flava**	GQ20.1	SpeciesID:20	MDA	93150894	41835127	0.09	2.09	0.65	2.00		1
**Pseudokeronopsis cf. flava**	GQ20.2	SpeciesID:20	MDA	71952360	41014909	0.05	3.10	0.74	5.00		1
**Pseudokeronopsis cf. flava**	GY20.2	SpeciesID:20	MALBAC	68447346	36894927	0.01	2.44	0.67	1.00		1
**Pseudokeronopsis cf. flava**	GY20.3	SpeciesID:20	MALBAC	90495349	57484718	0.05	2.22	0.65	15.00	>1	
**Euplotes sp**	GQ21.2	SpeciesID:21	MDA	74794897	50556120	0.23	1.97	0.48	2.00		1
**Euplotes sp**	GQ21.3	SpeciesID:21	MDA	80132213	45758921	0.08	1.94	0.59	1.00	>1	
**Euplotes sp**	GY21.1	SpeciesID:21	MALBAC	82017585	53503673	0.04	2.45	0.59	2.00		1
**Euplotes sp**	GY21.2	SpeciesID:21	MALBAC	98824705	61475630	0.03	2.50	0.64	2.00		1
**Euplotes woodruffi**	GQ22.1	SpeciesID:22	MDA	87843889	82223850	0.84	1.47	0.62	5.00		1
**Euplotes woodruffi**	GQ22.3	SpeciesID:22	MDA	75952868	63704177	0.66	1.27	0.59	6.00	>1	
**Euplotes woodruffi**	GY22.1	SpeciesID:22	MALBAC	67284466	51787191	0.27	1.71	0.69	10.00		1
**Euplotes woodruffi**	GY22.2	SpeciesID:22	MALBAC	71197307	54538670	0.39	1.38	0.63	9.00		1
**Vorticella sp.**	GQ23.1	SpeciesID:23	MDA	82970530	23652207	0.03	2.23	0.61	3.00		1
**Vorticella sp.**	GQ23.2	SpeciesID:23	MDA	91858459	30270400	0.07	2.03	0.54	5.00		1
**Vorticella sp.**	GY23.1	SpeciesID:23	MALBAC	94527658	41070070	0.04	2.06	0.62	5.00		1
**Vorticella sp.**	GY23.3	SpeciesID:23	MALBAC	86445460	37080987	0.03	1.95	0.57	6.00	>1	

**Diophrys sp**	GQ24.1	SpeciesID:24	MDA	78252666	40765003	0.03	1.50	0.43	2.00		1
**Diophrys sp**	GQ24.3	SpeciesID:24	MDA	79872167	42267830	0.03	1.27	0.38	1.00	>1	
**Diophrys sp**	GY24.3	SpeciesID:24	MALBAC	84365487	52166727	0.03	2.01	0.52	4.00	>1	
**Zoothamnium sp.**	GQ25.1	SpeciesID:25	MDA	68143055	29423024	0.03	2.09	0.52	0.00		1
**Zoothamnium sp.**	GQ25.3	SpeciesID:25	MDA	71438598	32486172	0.03	2.13	0.52	0.00	>1	
**Zoothamnium sp.**	GY25.2	SpeciesID:25	MALBAC	78477008	47904175	0.02	2.24	0.56	1.00		1
**Zoothamnium sp.**	GY25.3	SpeciesID:25	MALBAC	90111831	52774346	0.02	2.39	0.63	1.00	>1	
**Paranassula sp.**	GQ26.1	SpeciesID:26	MDA	83631037	43606226	0.02	1.85	0.52	0.00		1
**Paranassula sp.**	GQ26.2	SpeciesID:26	MDA	78042293	43098766	0.04	1.38	0.33	0.00		1
**Paranassula sp.**	GQ26.3	SpeciesID:26	MDA	78743652	42975465	0.02	1.96	0.55	0.00	>1	
**Paranassula sp.**	GY26.1	SpeciesID:26	MALBAC	66839095	38681385	0.03	1.96	0.53	1.00		1
**Paranassula sp.**	GY26.2	SpeciesID:26	MALBAC	63751313	45277333	0.06	1.95	0.54	8.00		1
**Paranassula sp.**	GY26.3	SpeciesID:26	MALBAC	80709851	46602625	0.05	1.16	0.28	2.00	>1	
**Licnophora lyngbycola**	GQ27.2	SpeciesID:27	MDA	76646374	31770826	0.05	1.86	0.46	4.00		1
**Licnophora lyngbycola**	GQ27.3	SpeciesID:27	MDA	70781668	31747349	0.07	1.83	0.46	4.00	>1	
**Licnophora lyngbycola**	GY27.1	SpeciesID:27	MALBAC	77946355	39575872	0.02	1.96	0.52	2.00		1
**Licnophora lyngbycola**	GY27.3	SpeciesID:27	MALBAC	74082742	34410808	0.03	1.57	0.41	1.00	>1	
**Hemiamphisiella sp.**	GQ28.2	SpeciesID:28	MDA	80743185	37787596	0.23	2.02	0.60	14.00		1
**Hemiamphisiella sp.**	GQ28.3	SpeciesID:28	MDA	67076328	23876220	0.05	2.10	0.63	10.00	>1	
**Trachelostyla pediculiformis**	GQ29.2	SpeciesID:29	MDA	69055254	36539228	0.08	2.67	0.67	9.00		1
**Trachelostyla pediculiformis**	GQ29.3	SpeciesID:29	MDA	74783903	49603157	0.15	2.29	0.53	11.00	>1	
**Trachelostyla pediculiformis**	GY29.1	SpeciesID:29	MALBAC	84654471	47144318	0.03	2.50	0.59	5.00		1
**Trachelostyla pediculiformis**	GY29.2	SpeciesID:29	MALBAC	92203505	51375383	0.03	2.46	0.65	5.00		1
**Tintinnopsis radix**	GQ3.1	SpeciesID:3	MDA	74646140	50297387	0.30	1.27	0.46	18.00		1
**Tintinnopsis radix**	GY3.1	SpeciesID:3	MALBAC	69715129	42041375	0.09	1.73	0.54	22.00		1
**Tintinnopsis radix**	GY3.2	SpeciesID:3	MALBAC	71375311	45851687	0.03	2.66	0.63	21.00		1

**Condylostoma sp.**	GQ31.2	SpeciesID:31	MDA	86801317	29142473	0.02	1.93	0.49	0.00	1
**Condylostoma sp.**	GQ31.3	SpeciesID:31	MDA	65593095	23023331	0.02	2.09	0.51	0.00	>1
**Condylostoma sp.**	GY31.1	SpeciesID:31	MALBAC	83507014	37532588	0.02	1.84	0.51	0.00	1
**Condylostoma sp.**	GY31.2	SpeciesID:31	MALBAC	70306633	30322749	0.02	1.63	0.44	2.00	1
**Apocoleps sp.**	GQ32.3	SpeciesID:32	MDA	78596290	47706988	0.04	2.18	0.60	0.00	>1
**Apocoleps sp.**	GY32.1	SpeciesID:32	MALBAC	83903747	67376526	0.36	1.37	0.47	9.00	1
**Apocoleps sp.**	GY32.3	SpeciesID:32	MALBAC	86383806	65328879	0.32	1.46	0.48	15.00	>1
**Chaenea vorax**	GQ33.2	SpeciesID:33	MDA	87403118	56309059	0.25	1.81	0.61	14.00	1
**Chaenea vorax**	GY33.1	SpeciesID:33	MALBAC	90593465	63877380	0.12	2.14	0.66	20.00	1
**Chaenea vorax**	GY33.2	SpeciesID:33	MALBAC	86181035	51367031	0.18	1.91	0.65	15.00	1
**Loxophyllum sp.**	GY34.1	SpeciesID:34	MALBAC	75250504	43513038	0.03	1.84	0.53	4.00	1
**Loxophyllum sp.**	GY34.3	SpeciesID:34	MALBAC	94850854	60898831	0.02	2.08	0.59	3.00	>1
**Amphileptus aeschtae**	GQ35.2	SpeciesID:35	MDA	89934818	54923589	0.04	2.40	0.63	15.00	1
**Amphileptus aeschtae**	GQ35.3	SpeciesID:35	MDA	98473288	61473361	0.07	2.30	0.66	5.00	>1
**Amphileptus aeschtae**	GY35.2	SpeciesID:35	MALBAC	76840217	68314937	0.30	1.82	0.58	19.00	1
**Amphileptus aeschtae**	GY35.3	SpeciesID:35	MALBAC	93250077	71250585	0.34	2.19	0.68	8.00	>1
**Hartmannula sinica**	GY36.1	SpeciesID:36	MALBAC	118688000	78060344	0.03	2.32	0.64	4.00	1
**Hartmannula sinica**	GY36.3	SpeciesID:36	MALBAC	90975772	63037205	0.04	2.48	0.82	4.00	>1
**Gruberia uninucleat**	GY37.1	SpeciesID:37	MALBAC	102772668	59844975	0.02	2.36	0.65	5.00	1
**Thigmokeronopsis rubra**	GY38.1	SpeciesID:38	MALBAC	80618765	44694147	0.05	2.21	0.53	6.00	1
**Thigmokeronopsis rubra**	GY38.2	SpeciesID:38	MALBAC	76557256	40893009	0.04	2.24	0.54	4.00	1
**Condylostentor auriculatus**	GQ39.2	SpeciesID:39	MDA	107161081	48675846	0.01	1.78	0.46	4.00	1
**Condylostentor auriculatus**	GQ39.3	SpeciesID:39	MDA	95431998	42442112	0.01	1.74	0.45	0.00	>1
**Eutintinnus sp.**	GQ4.1	SpeciesID:4	MDA	73578475	46228598	0.10	2.24	0.71	20.00	1
**Eutintinnus sp.**	GY4.1	SpeciesID:4	MALBAC	67952390	44160626	0.02	2.53	0.63	22.00	1
**Eutintinnus sp.**	GY4.2	SpeciesID:4	MALBAC	74216292	46759298	0.02	2.35	0.59	8.00	1

**Sterkiella sp.**	GQ40.2	SpeciesID:40	MDA	97462130	17199453	0.00	2.62	0.82	0.00	1
**Sterkiella sp.**	GQ40.3	SpeciesID:40	MDA	90852352	17064381	0.00	2.01	0.64	1.00	>1
**Colpidium Colpoda**	GQ41.2	SpeciesID:41	MDA	89903430	34206975	0.03	1.88	0.66	2.00	1
**Colpidium Colpoda**	GQ41.3	SpeciesID:41	MDA	103918023	38646896	0.01	2.26	0.62	2.00	>1
**Didinium sp.**	GQ42.1	SpeciesID:42	MDA	129096601	81895476	0.02	2.27	0.73	6.00	1
**Didinium sp.**	GQ42.3	SpeciesID:42	MDA	113402872	71615828	0.02	2.13	0.64	3.00	>1
**Frontonia sp.**	GQ43.2	SpeciesID:43	MDA	94882108	14872232	0.01	2.27	0.59	1.00	1
**Frontonia sp.**	GQ43.3	SpeciesID:43	MDA	92064340	13080747	0.01	2.37	0.67	0.00	>1
**Stylonychia bifaria**	GQ44.1	SpeciesID:44	MDA	79981095	25122538	0.03	1.90	0.63	3.00	1
**Stylonychia bifaria**	GQ44.2	SpeciesID:44	MDA	86452849	25989675	0.03	2.48	0.75	2.00	1
**Dysteria derouxi**	GQ45.1	SpeciesID:45	MDA	69494777	29523346	0.02	1.79	0.53	0.00	1
**Dysteria derouxi**	GQ45.3	SpeciesID:45	MDA	83838459	30373281	0.02	2.26	0.65	1.00	>1
**Dysteria derouxi**	GY45.3	SpeciesID:45	MALBAC	81838025	53522388	0.10	2.09	0.66	11.00	>1
**Rubrioxytricha haematoplasma**	GQ46.1	SpeciesID:46	MDA	77062895	36527979	0.02	1.93	0.58	3.00	1
**Rubrioxytricha haematoplasma**	GQ46.2	SpeciesID:46	MDA	79141029	34265957	0.02	2.03	0.63	2.00	1
**Rubrioxytricha haematoplasma**	GY46.2	SpeciesID:46	MALBAC	76617935	23962811	0.06	2.31	0.67	18.00	1
**Rubrioxytricha haematoplasma**	GY46.3	SpeciesID:46	MALBAC	68493287	20256996	0.05	2.15	0.66	8.00	>1
**Uroleptus longicaudatus**	GQ47.2	SpeciesID:47	MDA	80860810	20561491	0.01	2.55	0.82	3.00	1
**Uroleptus longicaudatus**	GQ47.3	SpeciesID:47	MDA	74998153	23782385	0.02	1.28	0.44	2.00	>1
**Uroleptus longicaudatus**	GY47.1	SpeciesID:47	MALBAC	81437658	30364494	0.04	2.65	0.78	9.00	1
**Uroleptus longicaudatus**	GY47.2	SpeciesID:47	MALBAC	77639177	34449972	0.07	2.64	0.73	19.00	1
**Tachysoma pellionellum**	GQ48.1	SpeciesID:48	MDA	74347117	28495181	0.03	1.90	0.54	2.00	1
**Tachysoma pellionellum**	GQ48.3	SpeciesID:48	MDA	88843037	43268692	0.10	1.37	0.62	2.00	>1
**Tachysoma pellionellum**	GY48.2	SpeciesID:48	MALBAC	66866034	28407614	0.03	2.63	0.68	11.00	1
**Tachysoma pellionellum**	GY48.3	SpeciesID:48	MALBAC	71143316	29917364	0.02	2.57	0.66	9.00	>1
**Mylestoma anatinum**	GQ49.1	SpeciesID:49	MDA	81800812	65669467	0.48	1.53	0.58	2.00	1

**Mylestoma anatinum**	GQ49.3	SpeciesID:49	MDA	109854831	70339969	0.29	1.56	0.61	5.00	>1	
**Mylestoma anatinum**	GY49.2	SpeciesID:49	MALBAC	71937684	48591132	0.27	1.43	0.33	29.00		1
**Mylestoma anatinum**	GY49.3	SpeciesID:49	MALBAC	66584818	48729199	0.31	0.74	0.21	5.00	>1	
**Euplotes cf. woodruffi**	GQ5.1	SpeciesID:5	MDA	78863235	47438854	0.44	0.60	0.20	6.00		1
**Euplotes cf. woodruffi**	GY5.1	SpeciesID:5	MALBAC	76086239	49826050	0.06	0.78	0.22	2.00		1
**Euplotes cf. woodruffi**	GY5.2	SpeciesID:5	MALBAC	71812371	48088168	0.06	0.69	0.18	7.00		1
**Brachonella sp.**	GQ50.2	SpeciesID:50	MDA	94797938	87298103	0.70	1.60	0.63	11.00		1
**Brachonella sp.**	GQ50.3	SpeciesID:50	MDA	85664856	78389152	0.72	1.37	0.51	9.00	>1	
**Brachonella sp.**	GY50.1	SpeciesID:50	MALBAC	93593692	63097358	0.05	2.80	0.68	20.00		1
**Brachonella sp.**	GY50.2	SpeciesID:50	MALBAC	75999481	40962616	0.04	2.21	0.63	2.00		1
**Climacostomum sp.**	GQ51.1	SpeciesID:51	MDA	99459173	42291288	0.16	0.83	0.30	2.00		1
**Climacostomum sp.**	GQ51.2	SpeciesID:51	MDA	100068930	35202615	0.06	1.22	0.46	1.00		1
**Climacostomum sp.**	GY51.2	SpeciesID:51	MALBAC	76709676	35784912	0.03	1.77	0.69	2.00		1
**Climacostomum sp.**	GY51.3	SpeciesID:51	MALBAC	72212093	34914539	0.03	1.68	0.64	1.00	>1	
**Urostyla sp**	GQ52.1	SpeciesID:52	MDA	85737024	35258089	0.01	2.95	0.81	3.00		1
**Urostyla sp**	GQ52.2	SpeciesID:52	MDA	104706167	57619439	0.03	1.80	0.64	2.00		1
**Urostyla sp**	GY52.2	SpeciesID:52	MALBAC	87566242	37240924	0.11	1.47	0.64	2.00		1
**Urostyla sp**	GY52.3	SpeciesID:52	MALBAC	82293288	39518110	0.07	2.13	0.79	14.00	>1	
**Peritromus cf. kahli**	GQ53.2	SpeciesID:53	MDA	107028231	45434828	0.03	2.54	0.58	3.00		1
**Peritromus cf. kahli**	GQ53.3	SpeciesID:53	MDA	97318652	41500763	0.04	2.59	0.59	2.00	>1	
**Peritromus cf. kahli**	GY53.2	SpeciesID:53	MALBAC	71680674	38815600	0.02	2.39	0.58	0.00		1
**Peritromus cf. kahli**	GY53.3	SpeciesID:53	MALBAC	81007352	41271851	0.03	2.44	0.63	1.00	>1	
**Apoholosticha cf. sinica**	GQ54.1	SpeciesID:54	MDA	95540667	44859146	0.20	0.62	0.23	6.00		1
**Apoholosticha cf. sinica**	GQ54.2	SpeciesID:54	MDA	94479908	41617563	0.09	0.19	0.06	5.00		1
**Apoholosticha cf. sinica**	GY54.2	SpeciesID:54	MALBAC	78816618	46093244	0.03	1.46	0.56	9.00		1
**Apoholosticha cf. sinica**	GY54.3	SpeciesID:54	MALBAC	75181323	43478397	0.02	0.79	0.28	7.00	>1	

**Chlamydodon bourlandi**	GQ55.2	SpeciesID:55	MDA	76396015	31415593	0.13	1.72	0.66	2.00		1
**Chlamydodon bourlandi**	GQ55.3	SpeciesID:55	MDA	72461002	24132833	0.10	1.66	0.64	2.00	>1	
**Chlamydodon bourlandi**	GY55.1	SpeciesID:55	MALBAC	87685218	48407463	0.02	2.62	0.76	2.00		1
**Chlamydodon bourlandi**	GY55.2	SpeciesID:55	MALBAC	78692850	45161157	0.02	2.49	0.69	1.00		1
**Aspidisca leptaspis**	GQ56.2	SpeciesID:56	MDA	90136574	75223993	0.17	2.16	0.69	18.00		1
**Aspidisca leptaspis**	GQ56.3	SpeciesID:56	MDA	95698023	61567534	0.26	2.78	0.68	35.00	>1	
**Aspidisca leptaspis**	GY56.2	SpeciesID:56	MALBAC	84901985	54407264	0.03	2.67	0.59	11.00		1
**Aspidisca leptaspis**	GY56.3	SpeciesID:56	MALBAC	74956103	58100811	0.01	2.68	0.66	2.00	>1	
**Urosoma emarginata**	GQ57.2	SpeciesID:57	MDA	68817049	27283472	0.01	1.61	0.47	1.00		1
**Urosoma emarginata**	GQ57.3	SpeciesID:57	MDA	93881348	31387473	0.01	1.93	0.60	0.00	>1	
**Urosoma emarginata**	GY57.2	SpeciesID:57	MALBAC	81649133	30779096	0.01	2.07	0.59	2.00		1
**Urosoma emarginata**	GY57.3	SpeciesID:57	MALBAC	81705248	30160346	0.02	2.53	0.75	13.00	>1	
**Frontonia tchibisovae**	GQ58.1	SpeciesID:58	MDA	80420072	26731118	0.01	2.22	0.57	0.00		1
**Frontonia tchibisovae**	GQ58.3	SpeciesID:58	MDA	72025132	24474849	0.02	2.22	0.68	0.00	>1	
**Frontonia tchibisovae**	GY58.1	SpeciesID:58	MALBAC	76902726	48460270	0.17	2.45	0.67	16.00		1
**Frontonia tchibisovae**	GY58.2	SpeciesID:58	MALBAC	78112156	46131632	0.06	2.12	0.56	11.00		1
**Urostomides sp.**	GQ59.1	SpeciesID:59	MDA	79188996	56693765	0.41	1.64	0.67	4.00		1
**Urostomides sp.**	GQ59.2	SpeciesID:59	MDA	86377102	58956891	0.23	1.15	0.41	3.00		1
**Urostomides sp.**	GY59.1	SpeciesID:59	MALBAC	83775876	54020409	0.04	1.88	0.55	9.00		1
**Urostomides sp.**	GY59.2	SpeciesID:59	MALBAC	87685727	54265458	0.03	2.06	0.54	11.00		1
**Favella sp.**	GQ6.2	SpeciesID:6	MDA	107553981	94705299	0.32	1.60	0.47	13.00		1
**Favella sp.**	GY6.1	SpeciesID:6	MALBAC	78071126	47285635	0.04	2.62	0.66	10.00		1
**Favella sp.**	GY6.2	SpeciesID:6	MALBAC	85821217	48031928	0.04	2.51	0.60	11.00		1
**Hemicycliostyla franzi**	GQ60.1	SpeciesID:60	MDA	70629143	42671118	0.05	1.67	0.54	6.00		1
**Hemicycliostyla franzi**	GQ60.2	SpeciesID:60	MDA	76162318	46494052	0.03	1.33	0.43	6.00		1
**Hemicycliostyla franzi**	GY60.1	SpeciesID:60	MALBAC	80550661	38659681	0.01	1.80	0.49	1.00		1

**Hemicycliostyla franzi**	GY60.2	SpeciesID:60	MALBAC	96340939	49866476	0.01	2.22	0.68	3.00	1
**Campanella umbellaria**	GQ61.1	SpeciesID:61	MDA	81235978	51626222	0.03	2.17	0.55	10.00	1
**Campanella umbellaria**	GQ61.2	SpeciesID:61	MDA	66804958	43296397	0.04	2.10	0.54	8.00	1
**Amphorellopsis sp.**	GQ62.1	SpeciesID:62	MDA	69607052	50957595	0.31	1.38	0.41	30.00	1
**Opercularia sp.**	GQ63.2	SpeciesID:63	MDA	72055234	30383333	0.05	1.95	0.47	10.00	1
**Certesia quadrinucleata**	GQ64.1	SpeciesID:64	MDA	67934170	37264836	0.03	2.45	0.68	2.00	1
**Certesia quadrinucleata**	GQ64.2	SpeciesID:64	MDA	70335320	41129248	0.05	2.33	0.66	2.00	1
**Certesia quadrinucleata**	GY64.1	SpeciesID:64	MALBAC	145805004	81751714	0.10	3.25	0.88	17.00	1
**Certesia quadrinucleata**	GY64.3	SpeciesID:64	MALBAC	168459091	97073855	0.08	3.12	0.85	13.00	>1
**Blepharisma penardi**	GQ65.1	SpeciesID:65	MDA	81581178	11799074	0.01	2.21	0.55	0.00	1
**Blepharisma penardi**	GQ65.2	SpeciesID:65	MDA	68072282	9357063	0.01	2.08	0.52	0.00	1
**Climacostomum virens**	GQ66.2	SpeciesID:66	MDA	124467288	38054079	0.04	1.45	0.54	2.00	1
**Climacostomum virens**	GY66.2	SpeciesID:66	MALBAC	79450657	35992602	0.03	1.75	0.64	2.00	1
**Climacostomum virens**	GY66.3	SpeciesID:66	MALBAC	74726455	34337773	0.06	1.66	0.69	2.00	>1
**Climacostomum virens**	GQ66.3	SpeciesID:66	MDA	122056665	37780800	0.05	1.50	0.55	2.00	>1
**Heterometopus palaeformis**	GQ67.2	SpeciesID:67	MDA	89629740	84139535	0.14	0.89	0.39	2.00	1
**Heterometopus palaeformis**	GQ67.3	SpeciesID:67	MDA	115354539	104529281	0.01	1.75	0.78	0.00	>1
**Heterometopus palaeformis**	GY67.2	SpeciesID:67	MALBAC	180677530	126419828	0.28	2.41	0.76	31.00	1
**Heterometopus palaeformis**	GY67.3	SpeciesID:67	MALBAC	166880829	95557886	0.07	3.13	0.82	25.00	>1
**Schmidingerothrix sp.**	GQ68.1	SpeciesID:68	MDA	96271658	30942240	0.00	1.63	0.62	0.00	1
**Schmidingerothrix sp.**	GY68.2	SpeciesID:68	MALBAC	60552294	42546806	0.43	2.25	0.73	46.00	1
**Schmidingerothrix sp.**	GY68.3	SpeciesID:68	MALBAC	65731221	43361418	0.33	2.24	0.74	24.00	>1
**Schmidingerothrix sp.**	GQ68.2	SpeciesID:68	MDA	41156491	23526584	0.01	1.96	0.57	0.00	1
**Homalogastra sp.**	GQ69.2	SpeciesID:69	MDA	81222064	75901328	0.84	0.85	0.50	2.00	1
**Homalogastra sp.**	GQ69.3	SpeciesID:69	MDA	63391974	60978549	0.92	1.03	0.53	7.00	>1
**Homalogastra sp.**	GY69.1	SpeciesID:69	MALBAC	99668559	83241606	0.67	1.90	0.74	26.00	1

**Homalogastra sp.**	GY69.2	SpeciesID:69	MALBAC	82724960	66754584	0.63	1.66	0.66	29.00	1
**Euplotes cf. parawoodruffi**	GQ7.2	SpeciesID:7	MDA	77812213	68367100	0.80	1.20	0.46	13.00	1
**Euplotes cf. parawoodruffi**	GY7.1	SpeciesID:7	MALBAC	71893716	50803079	0.15	1.34	0.39	24.00	1
**Euplotes cf. parawoodruffi**	GY7.2	SpeciesID:7	MALBAC	79967599	53983781	0.10	1.18	0.36	20.00	1
**Limnoholosticha viridis nov.sp.**	GQ70.2	SpeciesID:70	MDA	86400185	29127780	0.02	0.81	0.28	2.00	1
**Limnoholosticha viridis nov.sp.**	GQ70.3	SpeciesID:70	MDA	80242953	24761211	0.01	0.82	0.32	1.00	>1
**Limnoholosticha viridis nov.sp.**	GY70.1	SpeciesID:70	MALBAC	68372464	25659583	0.07	1.84	0.66	8.00	1
**Limnoholosticha viridis nov.sp.**	GY70.2	SpeciesID:70	MALBAC	93585901	35008054	0.07	1.78	0.64	9.00	1
**Saprodinium sp.**	GY71.2	SpeciesID:71	MALBAC	183784924	177662256	0.37	0.82	0.25	8.00	1
**Saprodinium sp.**	GY71.3	SpeciesID:71	MALBAC	203920783	196709570	0.26	1.10	0.36	6.00	>1
**Spirostomum**	GQ8.2	SpeciesID:8_30	MDA	103099810	36417901	0.06	1.33	0.40	3.00	1
**Spirostomum**	GY8.1	SpeciesID:8_30	MALBAC	68192953	36150789	0.03	2.38	0.67	2.00	1
**Spirostomum**	GY8.2	SpeciesID:8_30	MALBAC	83408446	46715925	0.04	2.30	0.73	6.00	1
**Spirostomum**	GQ30.2	SpeciesID:8_30	MDA	89883646	44180237	0.02	2.41	0.57	0.00	1
**Spirostomum**	GQ30.3	SpeciesID:8_30	MDA	85911845	41959715	0.03	2.27	0.52	0.00	>1
**Spirostomum**	GY30.2	SpeciesID:8_30	MALBAC	89878895	49117172	0.01	2.48	0.61	0.00	1
**Spirostomum**	GY30.3	SpeciesID:8_30	MALBAC	83015004	44159420	0.02	2.27	0.56	0.00	>1
**Urocentrum**	GQ9.2	SpeciesID:9	MDA	105070477	93979190	0.45	2.09	0.61	89.00	1
**Urocentrum**	GY9.1	SpeciesID:9	MALBAC	78794355	50522544	0.10	1.89	0.64	30.00	1
**Urocentrum**	GY9.2	SpeciesID:9	MALBAC	79671877	58897045	0.35	1.80	0.65	48.00	1
**Anteholosticha monilata**	Anteholosticha_DA1	SpeciesID:Anteholosticha_DA1	MALBAC	97292532	53754319	0.02	1.93	0.51	11.00	1
**Apodileptus visscheri**	Apodileptus	SpeciesID:Apodileptus	MALBAC	91233498	61294717	0.16	2.57	0.61	55.00	1
**Caenomorpha medusula**	Caenomorpha_medusula	SpeciesID:Caenomorpha_medusula	MALBAC	95987284	64048137	0.10	2.34	0.52	87.00	1
**Chilodontopsis depressa**	GR1.DNA	SpeciesID:Chilodontopsis_depressa-DNA_hemolei	MALBAC	84167706	51861665	0.21	2.30	0.62	28.00	1
**Colpoda variabilis**	MDA17	SpeciesID:Colpoda_variabilis_LBR2017052001_MDA17	MDA	68422491	39783569	0.05	2.27	0.67	3.00	1
**Cothurnia sp.**	Cothurnia_D13	SpeciesID:Cothurnia_D13	MALBAC	76714918	65816306	0.58	2.44	0.65	55.00	1

**Deviata n. sp.**	Deviata_DD1	SpeciesID:Deviata_DD1	MALBAC	71210648	32662270	0.17	1.91	0.64	22.00	1
**Dexiostoma**	Dexiostoma	SpeciesID:Dexiostoma	MALBAC	63004201	15429400	0.01	2.03	0.61	2.00	1
**Diophrys scutum**	Diophrys_scutum	SpeciesID:Diophrys_scutum	MALBAC	68909742	59846386	0.44	2.74	0.69	86.00	1
**Lacrymaria sp.1**	MDA03	SpeciesID:Lacrymaria_BY201703100_MDA03	MALBAC	69282285	54209162	0.39	2.61	0.69	33.00	1
**Lacrymaria sp.2**	MDA05	SpeciesID:Lacrymaria_BY2017031006_MDA05	MALBAC	67286759	29992943	0.05	1.89	0.61	5.00	1
**Litonotus sp.**	MDA04	SpeciesID:Litonotus_BY_MDA04	MALBAC	69067232	47354471	0.13	1.25	0.60	1.00	1
**Metopus**	Metopus_D11	SpeciesID:Metopus_D11	MALBAC	75784839	54743038	0.26	2.24	0.52	116.00	1
**Neourostylopsis**	MDA16	SpeciesID:Neourostylopsis_ZTY2017032702_MDA16	MDA	66785873	49720133	0.56	0.76	0.35	3.00	1
**Omegastrombidium**	MDA02	SpeciesID:Omegastrombidium_WR2017031001_MDA02	MDA	67741782	39731669	0.04	2.07	0.72	7.00	1
**Phacodinium**	Phacodinium_D15	SpeciesID:Phacodinium_D15	MALBAC	70223874	33247247	0.02	1.65	0.40	1.00	1
**Stenia**	Stenia	SpeciesID:Stenia		71016540	20989674	0.03	1.83	0.49	4.00	1
**Stentor roeselii**	Stentor_DS1	SpeciesID:Stentor_DS1	MALBAC	88776443	24517115	0.14	1.61	0.64	15.00	1
**Strobilidium gyrans**	Strobilidium_gyrans_D12	SpeciesID:Strobilidium_gyrans_D12	MALBAC	87824526	71989987	0.42	2.75	0.60	90.00	1
**Trithigmostoma steini**	Trithigmostoma	SpeciesID:Trithigmostoma	MALBAC	56397578	26092340	0.02	2.03	0.54	4.00	1
**Uronychia binucleata**	Uronychia_binucleata	SpeciesID:Uronychia_binucleata	MALBAC	59027368	32472218	0.03	2.87	0.72	5.00	1

Table S2. Taxonomic and integrity information of 148 representative single genomic bins. samleName Count indicates the number of samples assembled for the bin; **RepresentBin ANI** represents the maximum ANI value of the genome as compared to the refence; **RepresentBin refID** indicates the ID of the largest ANI, and **RepresentBin refSpecies** indicates the name of the corresponding species.

ID	samleName Count	RepresentBin ANI	RepresentBin refID	RepresentBin refSpecies	RepresentBin type	RepresentBin completeness	RepresentBin contamination	RepresentBin GC	RepresentBin N50	RepresentBin size	CatBatlower	CatBatlineage phylum
0	3	99.0762	GCF_013374815.1	Shigella sonnei	Known	99.16	0.264	0.507	79332	4646780	Proteobacteria	Bacteria (superkingdom): 0.77
1	3	82.4365	GCF_003574485.1	Facilibium subflavum	Novel	87.64	0.574	0.333	113890	1093541	Proteobacteria	Fastidiosibacteraceae (family): 0.91
2	1	NA	NA	NA	Novel	85.46	2.992	0.391	6412	2207591	Bacteroidetes	Marinifilaceae (family): 0.65
3	1	99.4633	GCF_011684115.1	Rahnella aceris	Known	64.96	0.163	0.518	158168	2539051	Proteobacteria	Rahnella (genus): 0.31
4	3	NA	NA	NA	Novel	87.52	0	0.453	9874	1950983	Proteobacteria	Deltaproteobacteria (class): 0.91
5	2	81.3974	GCF_009938305.1	Fluviibacter phosphoraccumulans	Novel	91.96	0	0.544	79622	1563698	Proteobacteria	unclassified Rhodocyclaceae (no rank): 0.51
6	3	NA	NA	NA	Novel	75.96	4.537	0.355	5321	979007	Proteobacteria	Proteobacteria (phylum): 0.34
7	1	74.913	GCF_000287275.1	Candidatus Carsonella ruddii HC isolate Thao2000	Novel	85.59	0	0.286	142931	989368	Firmicutes	Firmicutes (phylum): 0.38
8	3	80.871	GCF_009938305.1	Fluviibacter phosphoraccumulans	Novel	75	1.724	0.571	9706	1707186	Proteobacteria	unclassified Rhodocyclaceae (no rank): 0.54
9	3	77.6537	GCF_003953955.1	Candidatus Aquarickettsia rohweri	Novel	90.59	0.299	0.31	5904	853820	Proteobacteria	Rickettsiales (order): 0.90
10	6	96.4297	GCF_000376545.2	Tritonibacter mobilis F1926	Known	89.31	0.706	0.589	25520	3929600	Proteobacteria	Rhodobacteraceae (family): 0.93
11	2	79.4572	GCF_009938305.1	Fluviibacter phosphoraccumulans	Novel	89.1	0.676	0.574	32685	1552936	Proteobacteria	unclassified Rhodocyclaceae (no rank): 0.53

12	1	96.418	GCF_008693705.1	Aeromonas veronii	Known	74.13	4.31	0.589	45183	4555931	Proteobacteria	Aeromonas (genus): 0.71
13	1	NA	NA	NA	Novel	57.98	0.574	0.356	25889	1800648	Proteobacteria	Gammaproteobacteria (class): 0.68
14	1	96.9805	GCF_017310215.1	Aeromonas hydrophila	Known	54.31	3.448	0.62	15220	3852457	Proteobacteria	Aeromonas (genus): 0.62
15	1	83.2196	GCF_000009365.1	Alcanivorax borkumensis SK2	Intermediate	97.45	0.991	0.584	85615	3565421	Proteobacteria	unclassified Alcanivorax (no rank): 0.59
16	1	95.9023	GCF_900115865.1	Donghicola eburneus	Known	63.79	1.724	0.589	77543	3341043	Proteobacteria	Donghicola eburneus (species): 0.66
17	3	NA	NA	NA	Novel	82.9	1.424	0.474	146549	4701803	Terrabacteria group	Cyanobacteria/Melainabacteria group (clade): 0.57
18	1	97.226	GCF_020783375.1	Pseudomonas chloritidismutans	Known	74.12	0.862	0.624	6264	2880935	Proteobacteria	Gammaproteobacteria (class): 0.53
19	6	94.0335	GCF_001612705.2	Pseudomonas yamanorum	Intermediate	98.05	1.017	0.609	39701	7057567	Proteobacteria	Pseudomonas (genus): 0.62
20	1	92.6113	GCF_004000275.1	Saccharospirillum alexandrii	Intermediate	63.79	3.448	0.568	5692	3578215	Proteobacteria	Saccharospirillum alexandrii (species): 0.43
21	1	82.2355	GCF_002374315.1	Halomonas nigrificans	Novel	55.48	0	0.55	13175	1893375	Proteobacteria	unclassified Halomonas (no rank): 0.39
22	1	98.748	GCF_000422425.1	Oceanospirillum beijerinckii DSM 7166	Known	82.17	1.347	0.48	6174	4008970	Proteobacteria	Oceanospirillum (genus): 0.93
23	1	96.524	GCF_001550135.1	Pseudoalteromonas shioyasakiensis	Known	51.25	0.168	0.41	100729	2424899	Proteobacteria	Pseudoalteromonas (genus): 0.52
24	1	98.4738	GCF_902498775.1	Marinobacter sp. EC-HK377	Known	57.75	0	0.573	4884	2947476	Proteobacteria	Marinobacter (genus): 0.87
25	3	NA	NA	NA	Novel	95.61	0.584	0.397	35527	2070783	Proteobacteria	Legionellaceae (family): 0.68
26	1	90.7086	GCF_000260985.4	Methylophaga nitratireducenticrescens	Intermediate	98.76	0.544	0.448	137908	2859985	Proteobacteria	Methylophaga (genus): 0.79
27	1	78.8244	GCF_017161445.1	Niveibacterium microcysteis	Novel	57.31	1.037	0.63	18905	2642738	Proteobacteria	Niveibacterium (genus): 0.39
28	1	82.1313	GCF_011046245.1	Rhizobium pseudoryzae	Novel	55.68	0	0.606	20428	4268987	Proteobacteria	Rhizobium (genus): 0.45

29	1	NA	NA	NA	Novel	56.08	1.895	0.337	60662	914206	Proteobacteria	Rickettsiaceae bacterium (species): 0.46
31	1	NA	NA	NA	Novel	79.59	0.574	0.395	86709	1642861	Proteobacteria	Gammaproteobacteria (class): 0.31
32	1	NA	NA	NA	Novel	89.03	2.58	0.405	21133	2010323	Proteobacteria	Deltaproteobacteria (class): 0.92
33	1	87.6484	GCF_000168975.1	Roseibium aggregatum IAM 12614	Intermediate	60.52	0.158	0.591	5082	3929807	Proteobacteria	Labrenzia (genus): 0.59
34	1	NA	NA	NA	Novel	72.99	0	0.56	8068	3125628	Chloroflexi	Chloroflexaceae (family): 0.44
35	1	NA	NA	NA	Novel	91.94	2.118	0.458	66937	5784708	Deinococcus-Thermus	Deinococci (class): 0.42
36	1	77.4228	GCF_016603745.1	Francisella philomiragia	Novel	53.59	0	0.306	45776	730191	Proteobacteria	Francisellaceae (family): 0.70
37	2	NA	NA	NA	Novel	73.29	0.025	0.423	81526	969397	Proteobacteria	Proteobacteria (phylum): 0.79
38	1	79.536	GCF_005876985.1	Rhodoferax bucti	Novel	62.19	4.867	0.603	5097	2836383	Proteobacteria	unclassified Rhodoferax (no rank): 0.32
39	4	NA	NA	NA	Novel	97.84	0	0.417	164266	2009606	Proteobacteria	Alphaproteobacteria (class): 0.43
41	1	NA	NA	NA	Novel	61.9	1.111	0.597	6061	3971911	Planctomycetes	Planctomycetes (phylum): 0.74
42	2	NA	NA	NA	Novel	91.41	0.609	0.363	258535	1192914	Proteobacteria	Proteobacteria (phylum): 0.34
43	1	NA	NA	NA	Novel	62.35	0	0.422	31615	1434861	Proteobacteria	Gammaproteobacteria (class): 0.39
44	1	NA	NA	NA	Novel	71.15	0.581	0.397	31732	1518459	unknow	Bacteria (superkingdom): 0.92
45	1	81.4855	GCF_004361915.1	Sediminibacterium goheungense	Novel	90.51	0	0.406	12327	3452221	Bacteroidetes	Chitinophagaceae (family): 0.88
46	1	NA	NA	NA	Novel	60.52	0.68	0.517	10443	2897054	Verrucomicrobia	Verrucomicrobiales (order): 0.49
47	1	NA	NA	NA	Novel	52.94	0	0.365	40276	902203	Bacteroidetes	Bacteroidetes (phylum): 0.39
48	1	NA	NA	NA	Novel	76.14	0.71	0.338	45593	863405	Proteobacteria	Rickettsiales bacterium (species): 0.33

49	2	98.6471	GCF_003003005.1	Mongoliibacter ruber	Known	99.24	0.02	0.392	90653	4754913	Bacteroidetes	Cyclobacteriaceae (family): 0.97
50	1	NA	NA	NA	Novel	92.98	0.609	0.38	173655	1252669	Proteobacteria	Proteobacteria (phylum): 0.33
51	1	98.5092	GCF_003815615.1	Chryseobacterium shandongense	Known	92.89	0	0.368	105846	3519886	Bacteroidetes	Weeksellaceae (family): 0.66
52	2	NA	NA	NA	Novel	94.01	4.32	0.409	38556	4473337	Acidobacteria	Acidobacteria (phylum): 0.80
53	1	NA	NA	NA	Novel	57.77	0	0.341	39513	2173177	Lentisphaerae	Lentisphaerae (phylum): 0.49
54	1	NA	NA	NA	Novel	93.16	0.427	0.472	158130	2046809	Proteobacteria	Betaproteobacteria (class): 0.57
55	2	82.5958	GCF_014641715.1	Polaromonas eurypsychrophila	Novel	96.1	0	0.627	104730	3426239	Proteobacteria	unclassified Polaromonas (no rank): 0.30
56	1	NA	NA	NA	Novel	92.9	1.29	0.519	190291	3142114	Proteobacteria	Syntrophorhabdus (genus): 0.34
57	2	74.8251	GCF_000287275.1	Candidatus Carsonella ruddii HC isolate Thao2000	Novel	63.38	0	0.285	21555	1220372	Proteobacteria	Deltaproteobacteria (class): 0.37
58	1	NA	NA	NA	Novel	57.85	2.8	0.625	9646	2949169	Spirochaetes	Spirochaetales bacterium (species): 0.38
59	2	75.2362	GCF_000287275.1	Candidatus Carsonella ruddii HC isolate Thao2000	Novel	95.4	0.8	0.292	130741	3222431	Spirochaetes	unclassified Spirochaetes (no rank): 0.58
60	1	81.3506	GCF_000260135.1	Thiothrix nivea DSM 5205	Novel	93.52	1.494	0.548	30374	4474577	Proteobacteria	Thiothrix (genus): 0.90
61	1	97.7677	GCF_900102315.1	Celeribacter baekdonensis	Known	83.76	1.01	0.579	7253	3097660	Proteobacteria	Rhodobacteraceae (family): 0.99
62	4	NA	NA	NA	Novel	81.53	0	0.585	9761	4519323	Planctomycetes	Planctomycetes (phylum): 0.78
63	1	NA	NA	NA	Novel	50.18	2.247	0.554	4102	3443374	Planctomycetes	Planctomycetaceae (family): 0.38
64	1	NA	NA	NA	Novel	87.09	1.29	0.548	47449	1545942	Proteobacteria	Deltaproteobacteria (class): 0.40
65	1	NA	NA	NA	Novel	91.02	0.675	0.426	20008	2022771	Chlamydiae	Chlamydiia (class): 0.62
67	1	82.791	GCF_001551835.1	Dechloromonas denitrificans	Novel	72.41	0	0.626	11543	1841885	Proteobacteria	Betaproteobacteria (class): 0.72
68	2	89.484	GCF_003336675.1	Halomonas montanilacus	Intermediate	89.62	0.646	0.626	20347	3506634	Proteobacteria	Halomonas (genus): 0.94
69	1	NA	NA	NA	Novel	60.35	0.66	0.399	5648	3807487	Bacteroidetes	Bacteroidetes (phylum): 0.73

70	2	84.4868	GCF_001595965.1	Polynucleobacter yangtzensis	Intermediate	93.26	0.233	0.452	100129	1618439	Proteobacteria	Polynucleobacter (genus): 0.57
71	1	NA	NA	NA	Novel	62.04	0.055	0.383	46172	1467705	Bacteroidetes	Flavobacteriales (order): 0.32
72	1	89.3543	GCF_012927165.1	Zoogloea dura	Intermediate	59.48	0	0.659	7846	2490521	Proteobacteria	unclassified Zoogloea (no rank): 0.74
73	1	NA	NA	NA	Novel	56.12	0	0.443	208112	2279844	Proteobacteria	Oligoflexales bacterium* (species): 0.61
74	1	95.9686	GCF_014641635.1	Aquaticitalea lipolytica	Known	89.39	4.794	0.315	7369	3764181	Bacteroidetes	Flavobacteriaceae (family): 0.84
75	2	NA	NA	NA	Novel	95.12	0.731	0.332	33908	2061220	Proteobacteria	Neisseriales (order): 0.55
76	2	99.5275	GCF_000376705.1	Cutibacterium acnes HL096PA1	Known	89.03	2.302	0.603	10665	2309482	Actinobacteria	Actinobacteria (class): 0.91
77	2	NA	NA	NA	Novel	64.11	1.428	0.397	6616	2213875	Bacteroidetes	Bacteroidetes bacterium (species): 0.37
78	2	78.2931	GCF_014644295.1	Emticicia aquatilis	Novel	91.25	0.744	0.383	18899	5620909	Bacteroidetes	Cytophagaceae (family): 0.74
79	1	NA	NA	NA	Novel	73.22	1.02	0.574	11986	4129647	Verrucomicrobia	Verrucomicrobiales (order): 0.40
80	2	84.9978	GCF_001571225.1	Hydrogenophaga palleronii NBRC 102513	Intermediate	98.98	0.934	0.662	27792	3788203	Proteobacteria	Comamonadaceae (family): 0.48
81	1	94.2838	GCF_003326475.1	Thalassospira xianhensis MCCC 1A02616	Intermediate	88.69	0.497	0.55	28682	4152117	Proteobacteria	Thalassospira (genus): 0.92
82	1	NA	NA	NA	Novel	62.08	3.933	0.563	5586	6388512	Planctomycetes	Planctomycetes (phylum): 0.65
83	2	79.5349	GCF_001870675.1	Roseinatronobacter thiooxidans	Novel	92.26	1.01	0.594	20543	3430232	Proteobacteria	Rhodobacteraceae (family): 0.63
84	1	84.9685	GCF_014204325.1	Arcicella rosea	Intermediate	57.78	0.454	0.351	18222	2818442	Bacteroidetes	Cytophagaceae (family): 0.80
85	1	86.3079	GCF_005871185.1	Limnobacter alexandrii	Intermediate	79.31	0	0.525	96558	3052245	Proteobacteria	Betaproteobacteria (class): 0.92
86	1	NA	NA	NA	Novel	58.18	0	0.472	12994	2509942	Bacteroidetes	Cytophagales (order): 0.52

87	3	NA	NA	NA	Novel	87.93	0.33	0.486	8741	5565693	Bacteroidetes	Bacteroidetes (phylum): 0.61
88	1	NA	NA	NA	Novel	57.68	0	0.331	73692	562165	Proteobacteria	Alphaproteobacteria (class): 0.49
89	1	NA	NA	NA	Novel	94.64	0.218	0.374	20264	4094229	Cyanobacteria	Oscillatoriales (order): 0.31
90	1	86.376	GCF_003346815.1	Halarcobacter bivalviorum	Intermediate	96.74	2.168	0.289	130597	2534916	Proteobacteria	Arcobacter group (no rank): 0.55
91	1	98.9382	GCF_900108915.1	Pseudospirillum japonicum	Known	58.62	0	0.454	41330	2052818	Proteobacteria	Pseudospirillum japonicum* (species): 0.95
92	1	88.8597	GCF_000473205.1	Pseudophaeobacter arcticus DSM 23566	Intermediate	59.4	4.933	0.577	4882	3666524	Proteobacteria	Rhodobacteraceae (family): 0.88
93	1	78.6798	GCF_014203095.1	Prosthecobacter vanneervenii	Novel	66.23	0	0.613	9909	4254402	Verrucomicrobi a	unclassified Verrucomicrobiaceae (no rank): 0.34
94	2	77.3511	GCF_913698045.1	Candidatus Megaira endosymbiont of Carteria cerasiformis	Novel	98.57	2.132	0.333	51924	1292482	Proteobacteria	Alphaproteobacteria bacterium (species): 0.72
95	2	77.2785	GCF_000953135.1	Legionella fallonii LLAP-10	Novel	81.1	0.381	0.385	57579	1921550	Proteobacteria	Legionella (genus): 0.64
96	1	83.275	GCF_003172875.1	Leucothrix pacifica	Intermediate	90.52	3.534	0.454	15605	4729698	Proteobacteria	Leucothrix pacifica (species): 0.64
149	5	NA	NA	NA	Novel	58.71	0	0.393	7474	942112	Firmicutes	Negativicutes (class): 0.42
98	1	NA	NA	NA	Novel	95.4	0.287	0.367	10157	1983086	Proteobacteria	Fastidiosibacteraceae (family): 0.86
99	1	78.2555	GCF_001458735.1	Candidatus Nitrospira nitrosa	Novel	91.36	2.777	0.546	79469	4335946	Nitrospirae	Nitrospira (genus): 0.65
100	1	NA	NA	NA	Novel	65.1	0.102	0.391	5226	1370881	Elusimicrobia	unclassified Elusimicrobia (no rank): 0.50
101	1	NA	NA	NA	Novel	74.61	0.434	0.416	16239	1429386	Proteobacteria	Caedimonas varicaedens* (species): 0.92
102	1	NA	NA	NA	Novel	69.37	1.123	0.29	128439	979498	Bacteria candidate phyla	unclassified Candidatus Gracilibacteria* (no rank): 0.82
103	1	78.4705	GCF_008806775.1	Flavobacterium luteum	Novel	98.93	0	0.337	138280	2365250	Bacteroidetes	Flavobacteriaceae (family): 0.39

104	1	NA	NA	NA	Novel	78.6	0.188	0.505	10293	3731267	Chloroflexi	Chloroflexaceae (family): 0.42
105	2	77.3349	GCF_003953955.1	Candidatus Aquarickettsia rohweri	Novel	62.63	0	0.306	12110	713251	Proteobacteria	Rickettsiales (order): 0.91
106	1	NA	NA	NA	Novel	54.02	0.561	0.326	6984	779613	unknow	Bacteria (superkingdom): 0.87
118	9	NA	NA	NA	Novel	91.61	0.806	0.52	73084	2947975	Proteobacteria	Syntrophorhabdus (genus): 0.34
108	1	78.4705	GCF_009708035.1	Hyphomicrobium album	Novel	52.02	0	0.636	24953	1750156	Proteobacteria	Hyphomicrobium (genus): 0.41
109	1	NA	NA	NA	Novel	55.17	0	0.385	52097	1845200	Lentisphaerae	Lentisphaerae (phylum): 0.81
110	1	NA	NA	NA	Novel	64.66	0	0.352	482068	724399	Firmicutes	Firmicutes (phylum): 0.69
111	1	98.7906	GCF_913698045.1	Candidatus Megaira endosymbiont of Carteria cerasiformis	Known	67.77	0.473	0.335	10863	760001	Proteobacteria	Alphaproteobacteria bacterium (species): 0.92
112	1	NA	NA	NA	Novel	51.92	0.142	0.358	6052	1473954	Proteobacteria	Proteobacteria (phylum): 0.95
113	1	NA	NA	NA	Novel	75.27	0.473	0.353	60735	910530	Proteobacteria	Rickettsiales (order): 0.40
114	1	NA	NA	NA	Novel	83.05	0	0.361	194990	773296	Proteobacteria	Alphaproteobacteria (class): 0.57
115	1	NA	NA	NA	Novel	71.31	0.707	0.482	6805	3254380	Cyanobacteria	Aphanocapsa (genus): 0.36
116	1	NA	NA	NA	Novel	61.78	0.574	0.352	68819	1909262	Proteobacteria	Gammaproteobacteria (class): 0.68
117	1	NA	NA	NA	Novel	67.34	0	0.406	50564	1377465	Proteobacteria	Gammaproteobacteria (class): 0.51
119	2	NA	NA	NA	Novel	70.94	2.027	0.471	82338	2386472	Verrucomicrobi a	Verrucomicrobia (phylum): 0.32
120	1	NA	NA	NA	Novel	56.15	1.013	0.461	9814	2184282	Lentisphaerae	Lentisphaerae (phylum): 0.39
121	1	NA	NA	NA	Novel	52.35	2.702	0.386	9651	1853870	Lentisphaerae	Lentisphaerae (phylum): 0.84
122	1	NA	NA	NA	Novel	81.35	0.141	0.475	82768	2431880	Deinococcus-Thermus	Deinococcus (genus): 0.59
123	1	NA	NA	NA	Novel	81.03	1.149	0.378	35902	1418798	Proteobacteria	Fastidiosibacteraceae (family): 0.76

124	1	NA	NA	NA	Novel	75.47	1.149	0.382	6826	1250224	Proteobacteria	unclassified Gammaproteobacteria (no rank): 0.31
125	2	78.7833	GCF_002837135.1	Macromonas bipunctata	Novel	95.64	0.146	0.577	25907	2098693	Proteobacteria	Burkholderiales (order): 0.57
126	1	NA	NA	NA	Novel	53.06	0	0.526	195184	1259507	Proteobacteria	Syntrophus (genus): 0.58
128	1	NA	NA	NA	Novel	51.61	0	0.325	36673	1700815	Bacteroidetes	Bacteroidetes (phylum): 0.46
129	1	NA	NA	NA	Novel	81.18	1.184	0.541	22405	2265026	Proteobacteria	Alcaligenaceae (family): 0.39
130	1	NA	NA	NA	Novel	55.89	0.474	0.39	591098	875040	Bacteria candidate phyla	unclassified Candidatus Dependentiae (no rank): 0.53
131	1	74.9511	GCF_000287275.1	Candidatus Carsonella ruddii HC isolate Thao2000	Novel	71.42	0	0.285	62147	845194	Proteobacteria	Alphaproteobacteria (class): 0.57
132	1	NA	NA	NA	Novel	83.37	0	0.419	43559	1640564	Proteobacteria	Proteobacteria (phylum): 0.31
133	1	NA	NA	NA	Novel	51.3	3.225	0.418	5022	1988993	Bacteroidetes	Bacteroidetes (phylum): 0.45
134	3	NA	NA	NA	Novel	65.65	1.524	0.41	11877	884418	Proteobacteria	Proteobacteria (phylum): 0.42
135	1	NA	NA	NA	Novel	74.24	2.777	0.34	14388	4399041	Spirochaetes	Leptospiraceae* (family): 0.42
136	1	NA	NA	NA	Novel	65.69	0	0.392	75160	890008	Proteobacteria	Legionellales bacterium (species): 0.80
138	1	NA	NA	NA	Novel	57.12	0	0.305	106207	1006444	Bacteroidetes	Bacteroidetes (phylum): 0.38
139	1	NA	NA	NA	Novel	93.58	0	0.453	56581	1886901	Chlamydiae	Simkaniaceae (family): 0.62
140	1	NA	NA	NA	Novel	82.26	1.036	0.362	35599	1134377	Proteobacteria	Proteobacteria (phylum): 0.34
141	1	NA	NA	NA	Novel	56.93	0	0.348	74054	914031	Bacteria candidate phyla	Microgenomates group (clade): 0.42
142	1	NA	NA	NA	Novel	59.64	0	0.346	10445	722730	Proteobacteria	Rickettsiales (order): 0.62
144	2	NA	NA	NA	Novel	70.44	0	0.282	296010	495358	Proteobacteria	Proteobacteria (phylum): 0.32

145	2	75.43	GCF_018200315.1	Bacteroidetes bacterium endosymbiont of Orzyaephilus surinamensis	Novel	76.89	0.675	0.234	126113	598932	Lentisphaerae	Lentisphaerae (phylum): 0.69
146	1	78.1963	GCF_000237205.1	Simkania negevensis Z	Novel	90.2	0.94	0.409	6236	1728168	Chlamydiae	Simkania (genus): 0.92
147	1	NA	NA	NA	Novel	99.08	0.067	0.504	112990	2256896	Proteobacteria	Betaproteobacteria (class): 0.47
148	2	NA	NA	NA	Novel	92.73	0.581	0.394	114593	1591792	Proteobacteria	Gammaproteobacteria (class): 0.37
150	1	75.8569	GCF_012570365.1	Enterobacteriaceae endosymbiont of Donacia clavipes	Novel	72.16	0	0.233	284225	286186	Proteobacteria	Rickettsiales (order): 0.63
151	2	NA	NA	NA	Novel	81.91	0.843	0.403	150919	1608356	Firmicutes	Firmicutes (phylum): 0.42
152	1	NA	NA	NA	Novel	55.89	0.947	0.299	29801	656349	Proteobacteria	Rickettsiales (order): 0.35
153	1	NA	NA	NA	Novel	72.5	0	0.311	316216	632246	Proteobacteria	Proteobacteria (phylum): 0.35
107	1	NA	NA	NA	Novel	51.65	0.675	0.435	37556	867124	Verrucomicrobi a	Verrucomicrobia bacterium (species): 0.57

Table S3. The host information of the known symbiont and cyanobacteria.

full_name	maxAbundance	SummaryHost	IsCyanobacteria	Obligated_symbiont_or_in_literature	literature_details
**k__Bacteria;p__Proteobacteria;c__Gammaproteobacteria;o__Chromatiales;f__Chromatiaceae;g__Thiodictyon;s__Candidatus Thiodictyon syntrophicum**	10742.7	Metopus sp. (n=1/1)	No	Yes	A microbial eukaryote with a unique combination of purple bacteria and green algae as endosymbionts
**k__Bacteria;p__Cyanobacteria;c__;o__Chroococcales;f__Microcystaceae;g__Microcystis;s__Microcystis aeruginosa**	1.00E+06	Aspidisca leptaspis (n=1/4); Certesia quadrinucleata (n=2/4); Diophrys sp. (n=2/3); Euplotes sp. (n=3/4); Hemicycliostyla franzi (n=1/4); Uronychia setigera (n=2/4)	Yes	Yes	A new pentaplex-nested PCR to detect five pathogenic bacteria in free living amoebae
**k__Bacteria;p__Candidatus Nealsonbacteria;c__;o__;f__;g__;s__Candidatus Nealsonbacteria bacterium**	1416.1	Urocentrum sp. (n=1/3)	No	Yes	Anaerobic endosymbiont generates energy for ciliate host by denitrification
**k__Bacteria;p__Candidatus Poribacteria;c__;o__;f__;g__;s__Candidatus Poribacteria bacterium**	3119.5	Strombidinopsis sp. (n=1/3); Urocentrum sp. (n=1/3)	No	Yes	Bacteria Isolated From the Antarctic Sponge Iophon sp. Reveals Mechanisms of Symbiosis in Sporosarcina, Cellulophaga, and Nesterenkonia
**k__Bacteria;p__Proteobacteria;c__Gammaproteobacteria;o__Legionellales;f__Coxiellaceae;g__Rickettsiella;s__Candidatus Rickettsiella isopodorum**	48945.8	Apodileptus visscheri (n=1/1); Coleps sp. (n=3/4); Loxodes sp. (n=2/3); Metopus sp. (n=1/1)	No	Yes	Candidate pathogenicity islands in the genome of Candidatus Rickettsiella isopodorum, an intracellular bacterium infecting terrestrial isopod crustaceans
**k__Bacteria;p__Proteobacteria;c__Alphaproteobacteria;o__Holosporales;f__Candidatus Paracaedibacteraceae;g__Candidatus Finniella;s__Candidatus Finniella inopinata**	994891.6	Brachonella sp. (n=1/4); Coleps sp. (n=1/4); Hemicycliostyla franzi (n=4/4); Loxodes sp. (n=1/3)	No	Yes	Candidatus Finniella (Rickettsiales, Alphaproteobacteria), Novel Endosymbionts of Viridiraptorid Amoeboflagellates (Cercozoa, Rhizaria)

**k__Bacteria;p__Proteobacteria;c__Alphaproteobacteria;o__Rickettsiales;f__Rickettsiaceae;g__Candidatus Megaira;s__Candidatus Megaira endosymbiont of Carteria cerasiformis**	864799.7	Colpoda variabilis (n=1/1); Euplotes woodruffi (n=4/4); Neourostylopsis sp. (n=1/1); Stentor roeselii (n=1/1); Tachysoma pellionellum (n=1/4)	No	Yes	Candidatus Megaira polyxenophila gen. nov., sp. nov.: Considerations on Evolutionary History, Host Range and Shift of Early Divergent Rickettsiae
**k__Bacteria;p__Chlamydiae;c__Chlamydiia;o__Parachlamydiales;f__Parachlamydiaceae;g__Candidatus Protochlamydia;s__Candidatus Protochlamydia naegleriophila**	1951.5	Epistylis sp. (n=1/2)	No	Yes	Candidatus Protochlamydia amoebophila, an endosymbiont of Acanthamoeba spp
**k__Bacteria;p__Proteobacteria;c__Gammaproteobacteria;o__Thiotrichales;f__Francisellaceae;g__Francisella;s__Francisella adeliensis**	1.00E+06	Kiitricha marina (n=1/3)	No	Yes	Detection of a Novel Subspecies of Francisella noatunensis as Endosymbiont of the Ciliate Euplotes raikovi
**k__Bacteria;p__Proteobacteria;c__Gammaproteobacteria;o__Thiotrichales;f__Francisellaceae;g__Francisella;s__Francisella sp.**	70449.9	Kiitricha marina (n=1/3); Tintinnopsis radix (n=1/3)	No	Yes	Detection of a Novel Subspecies of Francisella noatunensis as Endosymbiont of the Ciliate Euplotes raikovi
**k__Bacteria;p__Proteobacteria;c__Gammaproteobacteria;o__Thiotrichales;f__Francisellaceae;g__Francisella;s__Francisella sp. FSC1006**	63350.9	Chlamydodon bourlandi (n=1/4)	No	Yes	Detection of a Novel Subspecies of Francisella noatunensis as Endosymbiont of the Ciliate Euplotes raikovi
**k__Bacteria;p__Proteobacteria;c__Gammaproteobacteria;o__Thiotrichales;f__Francisellaceae;g__Francisella;s__Francisella uliginis**	8564.3	Licnophora lyngbycola (n=1/4)	No	Yes	Detection of a Novel Subspecies of Francisella noatunensis as Endosymbiont of the Ciliate Euplotes raikovi
**k__Bacteria;p__Proteobacteria;c__Epsilonproteobacteria;o__Campylobacterales;f__Arcobacteraceae;g__Arcobacter;s__Arcobacter sp.**	3960.1	Diophrys scutum (n=1/1)	No	Yes	Environmental Breviatea harbour mutualistic Arco_x0002_bacter epibionts.
**k__Bacteria;p__Proteobacteria;c__Betaproteobacteria;o__Burkholderiales;f__Comamonadaceae;g__Candidatus Symbiobacter;s__Candidatus Symbiobacter mobilis**	8995.5	Spirostomum sp. (n=1/7)	No	Yes	Genomic analysis reveals key aspects of prokaryotic symbiosis in the phototrophic consortium Chlorochromatium aggregatum

**k__Bacteria;p__Proteobacteria;c__Alphaproteobacteria;o__Holosporales;f__Caedimonadaceae;g__Caedimonas;s__Caedimonas varicaedens**	694624.6	Climacostomum virens (n=4/4); Coleps sp. (n=1/4); Spirostomum sp. (n=3/7)	No	Yes	Holosporales
**k__Bacteria;p__Proteobacteria;c__Alphaproteobacteria;o__Holosporales;f__Candidatus Paracaedibacteraceae;g__;s__Candidatus Paracaedibacteraceae bacterium 'Lake Konstanz'**	190586.1	Anteholosticha monilata (n=1/1); Euplotes cf. parawoodruffi (n=1/3); Euplotes woodruffi (n=1/4); Urostyla sp. (n=1/4)	No	Yes	Holosporales
**k__Bacteria;p__Proteobacteria;c__Alphaproteobacteria;o__Rhodospirillales;f__Rhodospirillaceae;g__Candidatus Endolissoclinum;s__Candidatus Endolissoclinum sp. TMED37**	8781.6	Amphorellopsis sp. (n=1/1); Eutintinnus sp. (n=1/3); Strombidinopsis sp. (n=1/3)	No	Yes	Host Control of Symbiont Natural Product Chemistry in Cryptic Populations of the Tunicate Lissoclinum patella
**k__Bacteria;p__Proteobacteria;c__Alphaproteobacteria;o__Hyphomicrobiales;f__Devosiaceae;g__Devosia;s__Devosia sp. I507**	319940.1	Euplotes cf. parawoodruffi (n=1/3); Pleuronema sp. (n=2/3)	No	Yes	Identification of the bacterial endosymbionts of the marine ciliate Euplotes magnicirratus (Ciliophora, Hypotrichia) and proposal of Candidatus Devosia euplotis
**k__Bacteria;p__Proteobacteria;c__Gammaproteobacteria;o__Legionellales;f__Coxiellaceae;g__Candidatus Berkiella;s__Candidatus Berkiella aquae**	85915.4	Condylostentor auriculatus (n=1/2)	No	Yes	Infection and nuclear interaction in mammalian cells by Candidatus Berkiella cookevillensis, a novel bacterium isolated from amoebae
**k__Bacteria;p__Firmicutes;c__;o__;f__;g__;s__endosymbiont 'TC1' of Trimyema compressum**	319188.2	Plagiopyla nasuta (n=1/2)	No	Yes	Morphology and Phylogeny of a New Species of Anaerobic Ciliate, Trimyema finlayi n. sp., with Endosymbiotic Methanogens

Table S4. 18 pairs of biological replicates were tested for bacterial consistency. Number Of Dominate bacteira = 2 indicates that the dominate taxon has changed, and = 1 indicates that the dominate taxon has not changed.

PairedID	Sample1	Sample2	NumberOfDominateBacteira	pearson_cor
GQ22	GQ22.1	GQ22.3	1	0.999483714
GQ23	GQ23.1	GQ23.2	1	0.991507504
GQ42	GQ42.1	GQ42.3	1	0.994949162
GQ54	GQ54.1	GQ54.2	1	0.964121461
GQ59	GQ59.1	GQ59.2	1	0.969622152
GY19	GY19.2	GY19.3	1	0.999807209
GY22	GY22.1	GY22.2	1	0.973053189
GY23	GY23.1	GY23.3	1	0.998939498
GY29	GY29.1	GY29.2	1	0.986159304
GY34	GY34.1	GY34.3	1	0.969371921
GY7	GY7.1	GY7.2	1	0.990581237
GY9	GY9.1	GY9.2	1	0.998729222
GQ12	GQ12.1	GQ12.2	2	-0.378830798
GY3	GY3.1	GY3.2	2	-0.129132495
GY50	GY50.1	GY50.2	2	0.06747879
GY57	GY57.2	GY57.3	2	-0.379054331
GY59	GY59.1	GY59.2	2	0.256232121
GY69	GY69.1	GY69.2	2	0.309244486
